# Virally encoded connectivity transgenic overlay RNA sequencing (VECTORseq) defines projection neurons involved in sensorimotor integration

**DOI:** 10.1016/j.celrep.2021.110131

**Published:** 2021-12-21

**Authors:** Victoria Cheung, Philip Chung, Max Bjorni, Varvara A. Shvareva, Yesenia C. Lopez, Evan H. Feinberg

**Affiliations:** 1 Department of Anatomy, University of California, San Francisco, San Francisco, CA 94158, USA; 2 Tetrad Graduate Program, University of California, San Francisco, San Francisco, CA 94158, USA; 3 Department of Anesthesiology & Pain Medicine, University of Washington, Seattle, WA 98195, USA; 4 Kavli Institute for Fundamental Neuroscience, University of California, San Francisco, San Francisco, CA 94158, USA; 5 These authors contributed equally; 6 Lead contact

## Abstract

Behavior arises from concerted activity throughout the brain. Consequently, a major focus of modern neuroscience is defining the physiology and behavioral roles of projection neurons linking different brain areas. Single-cell RNA sequencing has facilitated these efforts by revealing molecular determinants of cellular physiology and markers that enable genetically targeted perturbations such as optogenetics, but existing methods for sequencing defined projection populations are low throughput, painstaking, and costly. We developed a straightforward, multiplexed approach, virally encoded connectivity transgenic overlay RNA sequencing (VECTORseq). VECTORseq repurposes commercial retrogradely infecting viruses typically used to express functional transgenes (e.g., recombinases and fluorescent proteins) by treating viral transgene mRNA as barcodes within single-cell datasets. VECTORseq is compatible with different viral families, resolves multiple populations with different projection targets in one sequencing run, and identifies cortical and subcortical excitatory and inhibitory projection populations. Our study provides a roadmap for high-throughput identification of neuronal subtypes based on connectivity.

## In brief

Neurons with different axonal projection targets and distinct molecular and functional properties are intermingled throughout the brain. Cheung et al. establish a high-throughput method, VECTORseq, to transcriptionally define projection neurons. The method reimagines transgenes expressed by widely used retrogradely infecting viruses as multiplexed RNA barcodes that are detected in single-cell sequencing.

## Graphical abstract

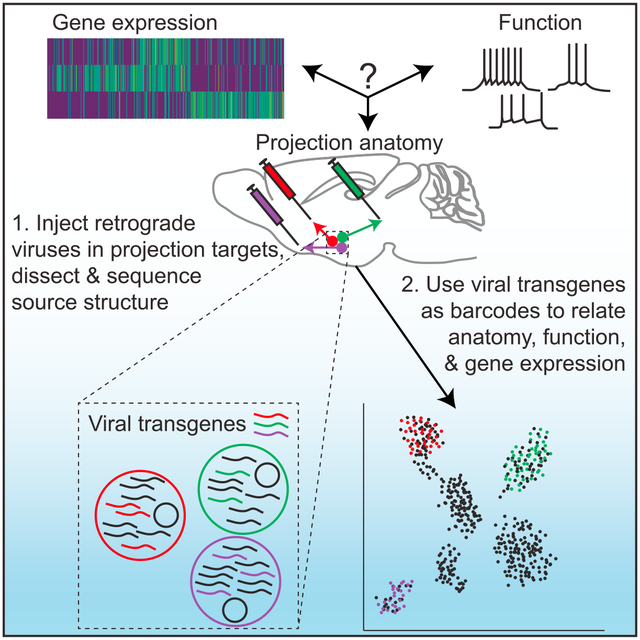

## INTRODUCTION

Functionally and molecularly diverse projection neurons with distinct targets are intermingled in most brain areas. For example, primary visual cortex contains functionally distinct populations that project to higher visual cortical areas, contralateral cortex, and subcortical targets such as striatum, thalamus, and superior colliculus; and substantia nigra pars reticulata in midbrain harbors subtypes that project to 39 target structures and differ in neurotransmitters released, response tuning, and intrinsic excitability (Antal et al., 2014; Jiang et al., 2003; Kim et al., 2015; Lur et al., 2016; McElvain et al., 2021; Poulin et al., 2016). A central goal of neuroscience is deciphering the properties and behavioral functions of the myriad projection neuron subtypes in the brain. Single-cell RNA sequencing (RNA-seq) technologies offer insights into the physiology of each identified neuronal population and molecular markers that could be used for targeted monitoring and manipulations during behavior. However, a challenge in interpreting single-cell RNA-seq datasets is to link populations identified through RNA expression to anatomy, connectivity, and circuit properties, a conundrum known as a “correspondence problem” (Lein et al., 2017).

One approach to this correspondence problem is to sequence a tissue, identify markers for many different cell types, and perform viral anterograde tracing in a panel of transgenic mice expressing Cre recombinase (Ding et al., 2020). This is a powerful, systematic approach, but entails obtaining or generating Cre transgenic mice for each population and is mouse and labor intensive. Therefore, in recent years, several approaches have been developed to focus transcriptional profiling on projection populations of interest. Many exploit dyes or viruses that are injected into the target structure, internalized at axon terminals, and trafficked retrogradely to label cell bodies in the source structure (Wickersham and Feinberg, 2012). In one approach, retrogradely labeled cells are isolated and sequenced (Tasic et al., 2018). In another, Patch-seq, cells are targeted for intracellular recording before the cellular contents are aspirated and sequenced (Cadwell et al., 2016; Fuzik et al., 2016). Both approaches are laborious, requiring separate rounds for each projection target and population of interest and additional steps for selective isolation of labeled cells, and costly, because each projection target is sequenced separately. A third sequencing-based approach, retroTRAP (translating ribosome affinity purification), relies on retrograde viruses to express tagged ribosomal subunits in projection neurons followed by immunoprecipitation of tagged mRNA (Ekstrand et al., 2014). Because this method does not resolve single cells, it obscures heterogeneity within projection populations; moreover, it must be performed on just one projection target at a time. A recently described sequencing-based method, multiplexed analysis of projections by sequencing (MAPSeq), traces connectivity anterogradely (Kebschull et al., 2016). A structure of interest is infected with Sindbis virus encoding RNA barcodes that are trafficked into axons and detected by RNA-seq of target structures. MAPseq can be combined with *in situ* sequencing of starter cells to identify their Sindbis-encoded barcodes and fluorescence *in situ* hybridization (FISH) to assign these starter cells to particular cell types (Chen et al., 2019). This method, barcoded anatomy resolved by sequencing (BARseq), allows the identification of multiple projection populations at once but requires additional steps and specialized equipment, including for *in situ* sequencing. Moreover, a prerequisite for BARseq is the use of standard single-cell sequencing to identify candidate markers for FISH, and Sindbis virus is highly toxic and rapidly disrupts cellular transcription, requiring calibration for every experiment. Thus, there is a pressing need for a means of simple, multiplexed transcriptional profiling of myriad projection cell types in the transcriptional “ground state” in a single sequencing experiment without specialized additional equipment. Such an approach would lower barriers to access, increase throughput, reduce the numbers of animals used per experiment, expedite experiments, and reduce costs.

We reasoned that retrogradely infecting viruses that are widely used to label and isolate projection populations (e.g., with fluorophores) could be repurposed as delivery systems for mRNA barcodes that are directly detected in single-cell sequencing datasets. This would enable multiplexed identification of projection cell types in one sequencing run without additional equipment. Here, we describe this approach, virally encoded connectivity transgenic overlay RNA sequencing (VECTORseq). We show that virally encoded transcripts delivered via retrograde infection are robustly detected by single-cell sequencing, readily distinguished from closely related isoforms, and found selectively in the expected populations in primary visual cortex. We then apply VECTORseq to multiple subcortical structures for the multiplexed identification of both known and additional projection populations in the transcriptional ground state. We thus establish a straightforward, high-throughput method to transcriptionally profile projection populations and uncover subcortical cell types involved in sensorimotor integration.

## RESULTS

**Figure 1.**
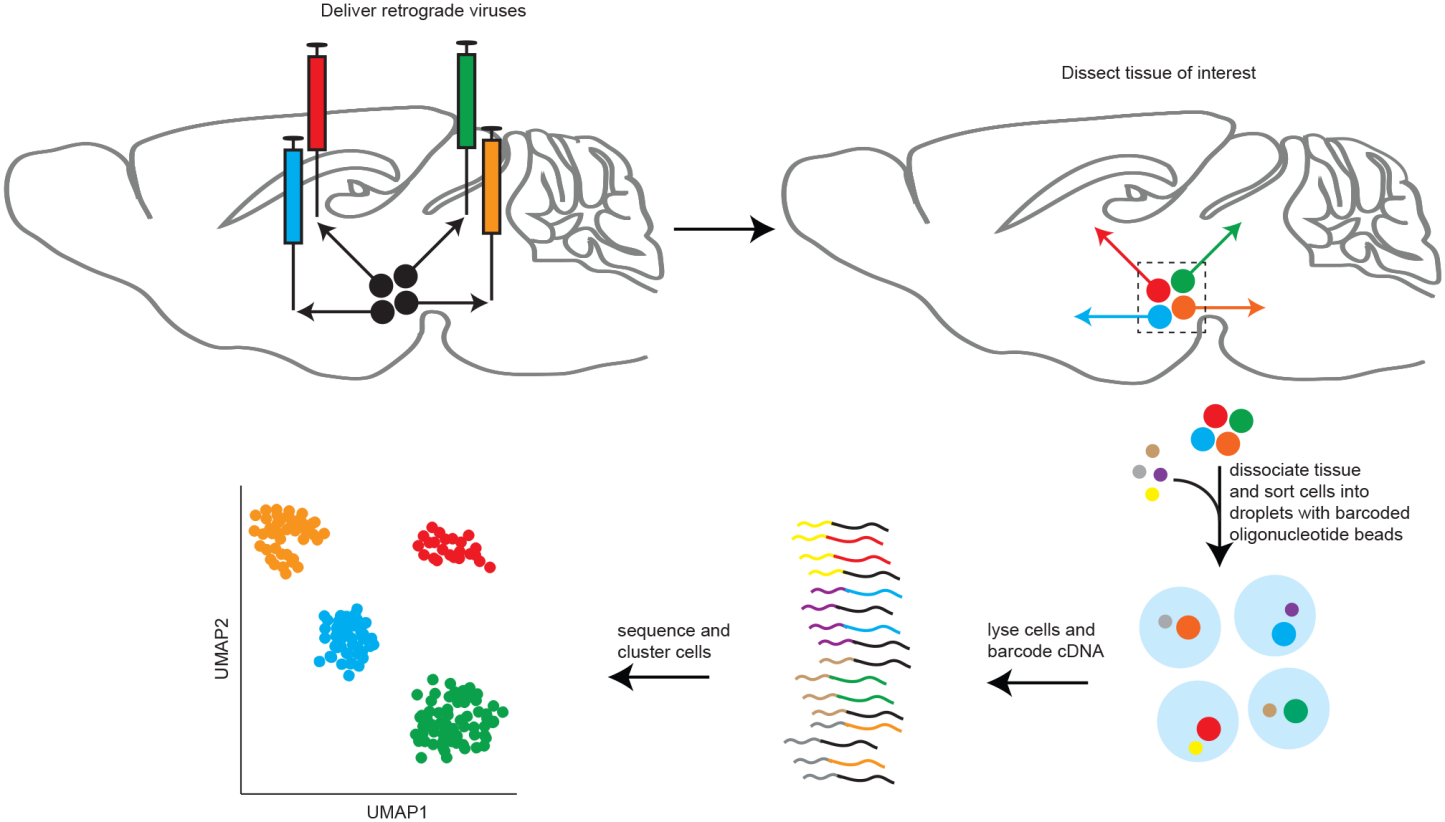
Schematic of VECTORseq Injecting retrogradely infecting viruses (colored syringes) into brain structures (sagittal section of mouse brain in this schematic) targeted by different projection neurons (directed arrows) from a single structure of origin will label each with unique virally encoded RNA barcodes. Following standard single-cell sequencing methods and analysis, the expression of viral barcodes can be overlaid to assign each cluster to its projection target.

VECTORseq repurposes widely used retrogradely infecting viruses that express transgenes such as recombinases and fluorophores by treating transgene mRNA as barcodes to overlay anatomy on single-cell sequencing data. For example, if we inject structure A with a retrogradely infecting virus encoding green fluorescent protein (GFP) and structure B with retrogradely infecting virus encoding Cre recombinase, cells that project to structure A will express *GFP* mRNA, whereas cells that project to structure B will express *Cre* mRNA (Figure 1). Thus, in a single-cell sequencing dataset of structure C, cells expressing *Cre* or *GFP* mRNA can be identified as projecting to structure A or B, respectively.

### Validation of VECTORseq in primary visual cortex

**Figure 2.**
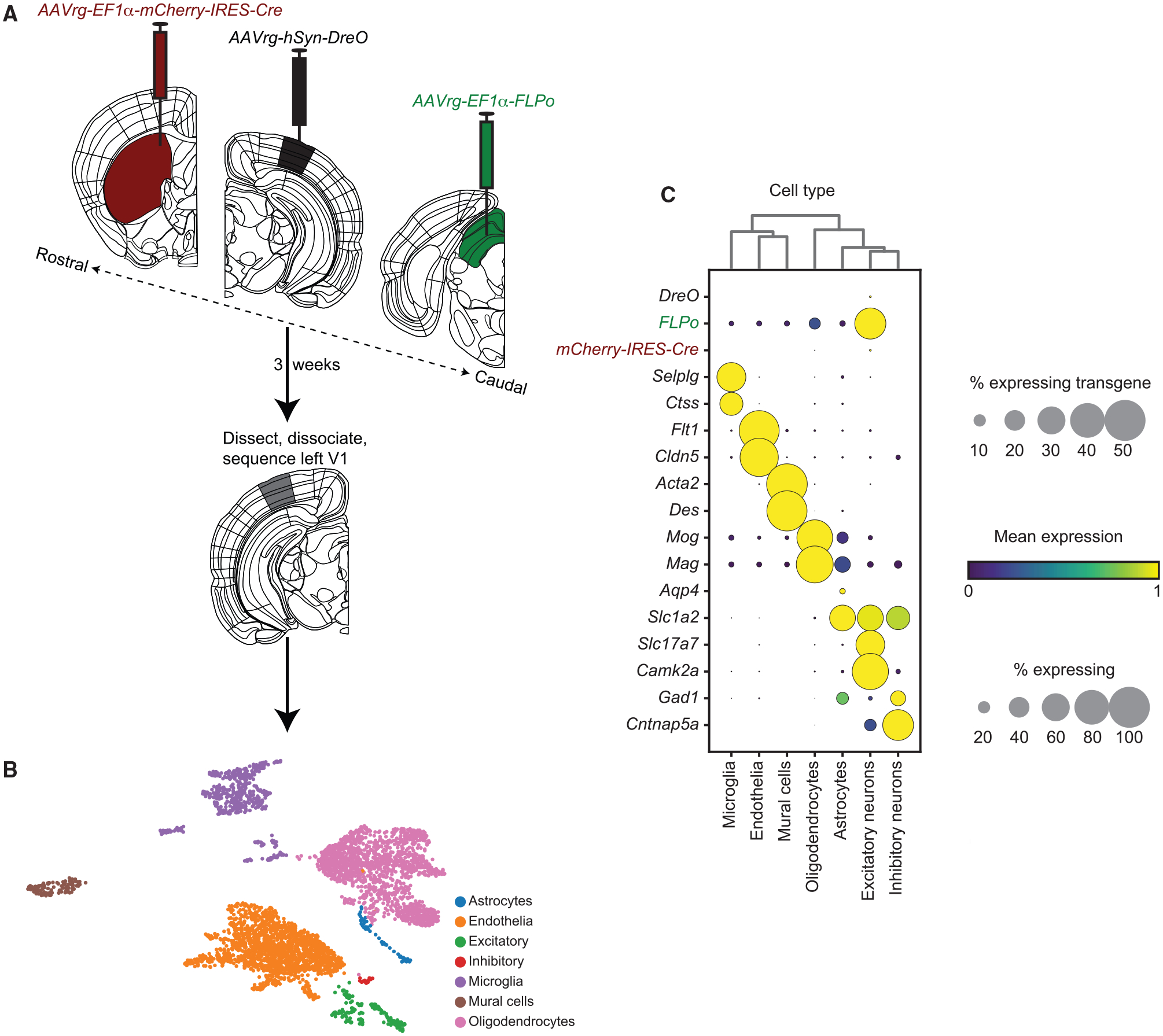
VECTORseq of V1 projection neurons (A) Retrograde viruses were injected into left SC, left striatum, and right V1. To mark injection sites in SC and contralateral V1, dilute *AAV1-hSyn-TurboRFP* was co-injected (not illustrated). As a fiducial for microdissection, left V1 was also injected with *AAV1-CAG-FLEX-tdTomato* and *AAV1-EF1α-fDIO-EYFP* (not illustrated). Three weeks later, left V1 was dissected, cells were dissociated, and single-cell sequencing was performed. Anatomical schematics from the Allen Brain Reference Atlas (Wang et al., 2020). (B) UMAP plot illustrating different major cell types in this dataset. (C) Enriched and differentially expressed genes in major cortical cell types and viral transgenes. Note the different scales for transgenes and endogenous genes.

We tested the feasibility of VECTORseq on projection populations in primary visual cortex (V1). These populations offered benchmarks because they had been transcriptionally profiled using fluorescence to isolate retrogradely labeled cells and are robustly infected by the retrogradely infecting adeno-associated virus (AAV) serotype AAVrg (Tasic et al., 2018; Tervo et al., 2016). AAVrg appealed because our goal was to define transcriptional ground states and AAV is thought to preserve cellular physiology (Haggerty et al., 2019) and commercial sources offer AAVrg encoding diverse transgenes (e.g., *GFP, tdTomato, Cre*). In five mice, we injected off-the-shelf AAVrg encoding distinct transgenes into three V1 projection targets: *AAVrg*-*EF1α-mCherry-IRES-Cre* in left striatum; *AAVrg-EF1α*-*FLPo* in left superior colliculus (SC); and *AAVrg-hSyn-Dre* in right (contralateral) V1 (Figure 2A) (Kim et al., 2015; Lur et al., 2016; Tasic et al., 2018; Tervo et al., 2016). To visualize injection sites of viruses encoding non-fluorescent transgenes (FLPo and Dre), we included *AAV1-hSyn-TurboRFP*; because AAV1 can traffic retrogradely, we diluted this virus (Tervo et al., 2016) (Figure S1). We injected left V1 with AAV1 encoding a Cre-dependent tdTomato (*CAG-FLEX-tdTomato*) and a FLP-dependent yellow fluorescent protein (*EF1α-fDIO-EYFP*) as fiducials for microdissection and, if necessary, signal amplification for the *mCherry-IRES-Cre* and *FLPo*, respectively (Figure 2A). Three weeks later, we dissected left V1, isolated single cells, processed cells using the 10x Chromium system, and performed paired-end sequencing using the Illumina NextSeq platform (Figure 2A). We used the Chromium 5′ kit because many commercial AAVs incorporate the 3′ woodchuck hepatitis virus posttranscriptional regulatory element (WPRE) to boost transgene expression (Wang et al., 2016). For this pilot experiment, sequencing depth was relatively shallow and relatively few cells were sequenced.

We detected 21,702,272 reads in 4,167 cells; 15 reads aligned to *mCherry-IRES-Cre*; 8,007 reads aligned to *FLPo*; and 1 read aligned to *Dre*. Within cells expressing the viral transgenes, expression was as strong as that of many common marker genes (Table S1). In addition, 114 reads aligned to the diluted *TurboRFP* virus injected in SC and contralateral V1. To determine the specificity of VECTORseq, we added to our library the sequence of a different common *Cre* isoform; these isoforms have codon substitutions that render them 74% identical (Figure S2). Whereas 15 reads aligned to *mCherry-IRES-Cre*, none aligned to the other *Cre*. Thus, VECTORseq is sufficiently sensitive to detect retrograde transcripts and sufficiently specific to discriminate homologous transgenes in single-cell datasets. Although robust detection of the *Cre* and *FLP* obviated the need for the reporters for signal amplification, we detected abundant *tdTomato* and *EYFP* reads (33,983 reads in 769 cells and 10,045 reads in 350 cells, respectively). This widespread *tdTomato* and *EYFP* expression likely reflected leaky antisense transcription or recombination during plasmid production (Fischer et al., 2019).

We used the Leiden algorithm to cluster cells and uniform manifold approximation and projection (UMAP) to visualize and annotate clusters based on the expression of known marker genes, separating inhibitory and excitatory neurons, endothelia, and glia (Figure 2B) (Chamling et al., 2021; Chen et al., 2020; Hammond et al., 2019; Hasel et al., 2017; He et al., 2016; Traag et al., 2019; Yao et al., 2021). Because previous studies of sorted and retrogradely labeled cortical projections to SC, striatum, and contralateral cortex found a variety of mostly excitatory neurons in cortical layers 2–6, we predicted that transgenes would be enriched in the excitatory cluster (Tasic et al., 2018). Indeed, all of the retrograde transgenes were enriched in excitatory neurons (Figure 2C). These data indicate the feasibility of VECTORseq—transgenes delivered by retrogradely infecting viruses are detected in single-cell sequencing datasets in the correct cell types.

### Application of VECTORseq to SC projection populations

**Figure 3.**
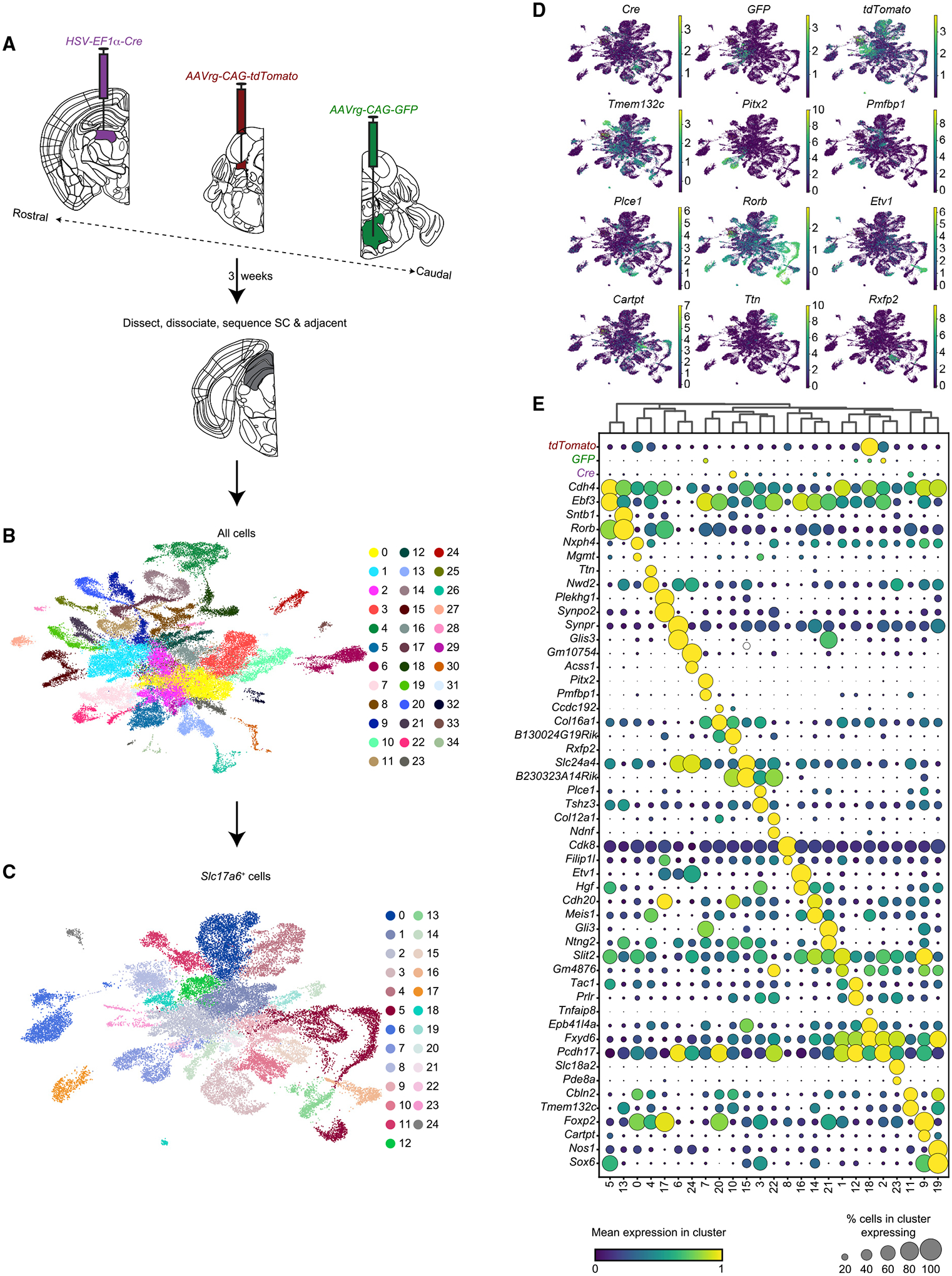
VECTORseq of SC (A) Retrograde viruses were injected into left LP, left CnF, and right PPRF. Three weeks later, left SC was dissected; nuclei were isolated, stained, and sorted according to *NeuN/Rbfox3* expression; and single-cell sequencing was performed. (B) UMAP plot of SC sequencing data and clustering. (C) UMAP plot of SC excitatory (*Slc17a6*^+^) neurons. (D) UMAP plots of SC excitatory neurons with expression of viral transgenes or example marker genes overlaid. (E) Enriched and differentially expressed genes in major SC excitatory cell types and distribution of retrograde viral transgenes.

We next applied VECTORseq to a subcortical structure, SC, which harbors both known and uncharacterized projection populations. We targeted two SC cell types that innervate the brain-stem: neurons that control orienting movements and innervate contralateral paramedian pontine reticular formation (PPRF, which includes medial portions of the caudal and oral pontine reticular nuclei, as well as paraabducens nucleus) and neurons that drive avoidance responses and innervate ipsilateral cuneiform nucleus (CnF) (Dean et al., 1989; May and Corbett, 2018; Sahibzada et al., 1986). A previous study labeled both with lenti-viruses, but we were unable to identify a commercial source (Isa et al., 2020). Therefore, we attempted to use AAVrg, injecting right PPRF with *AAVrg-CAG-GFP* and left CnF with *AAVrg-CAG-tdTomato* (Figures 3A and S3A). In addition, we targeted SC projections to thalamic lateral posterior nucleus (LP), the homolog of primate pulvinar. Anterograde tracing from SC labels multiple subdivisions of LP, including LPLR and LPMR, and studies in primates found that projections from, respectively, superficial and deep SC target the homologous regions of pulvinar (Benevento and Fallon, 1975; Gale and Murphy, 2014; Gharaei et al., 2020; Homman-Ludiye and Bourne, 2019). A serendipitously discovered Cre transgenic line, *Ntsr1-GN209*, labels wide-field (WF) cells in superficial SC that project to LPLR and are implicated in visual processing and fear responses, although endogenous Ntsr1 does not appear to be expressed in WF cells (Gale and Murphy, 2014; Gerfen et al., 2013). This Cre line has become a popular tool for studying the involvement of SC and LP in visual processing and behavior (Gale and Murphy, 2014, 2016; Hoy et al., 2019; Reinhard et al., 2019; Sans-Dublanc et al., 2021). However, the molecular and functional properties of the projection from deep SC to LPMR remain unknown. We therefore targeted LP to transcriptionally profile WF cells and this undefined LPMR-projecting population. Another retrograde virus reported to preserve cellular ground states, herpes simplex 1 (HSV-1), infects WF cells (Neve et al., 2005; Reinhard et al., 2019; Verlengia et al., 2017). Therefore, to test the applicability of VECTORseq to another widely used retrograde virus, we injected *HSV-Cre* at the border between LPLR and LPMR to label SC neurons projecting to each (Figures 3A and S3B).

Three weeks later, we dissected and dissociated dorsal midbrain, containing SC and adjacent structures, such as portions of periaqueductal gray and inferior colliculus, from 4 mice. Because sequencing neuronal nuclei has become popular, we determined the compatibility of VECTORseq with this approach, isolating nuclei expressing the neuronal marker *NeuN/Rbfox3* (Krishnaswami et al., 2016). We generated single-nucleus libraries using 10x Chromium 5′ kits and performed Illumina sequencing using the NovaSeq platform. These analyses revealed 628,122,200 reads corresponding to 54,537 cells. A total of 170,025 reads (in 11,377 cells) aligned to *AAVrg-CAG-tdTomato*, 5,270 reads (in 1,531 cells) aligned to *AAVrg-CAG-GFP*, and 15,861 reads (in 3,117 cells) aligned to *HSV-Cre*, showing the compatibility of VECTORseq with single-nucleus sequencing approaches and with viruses other than AAV. As in cortex, these viral transgenes were expressed at levels comparable to those of common marker genes such as *Rbfox3*, *Slc17a6*, and *Gad1* (Table S2). Importantly, although 15,861 reads aligned to the injected *Cre* isoform, none aligned to the *mCherry-IRES-Cre* used in the V1 experiment, further demonstrating the specificity of VECTORseq in discriminating similar transgenes.

**Figure 4.**
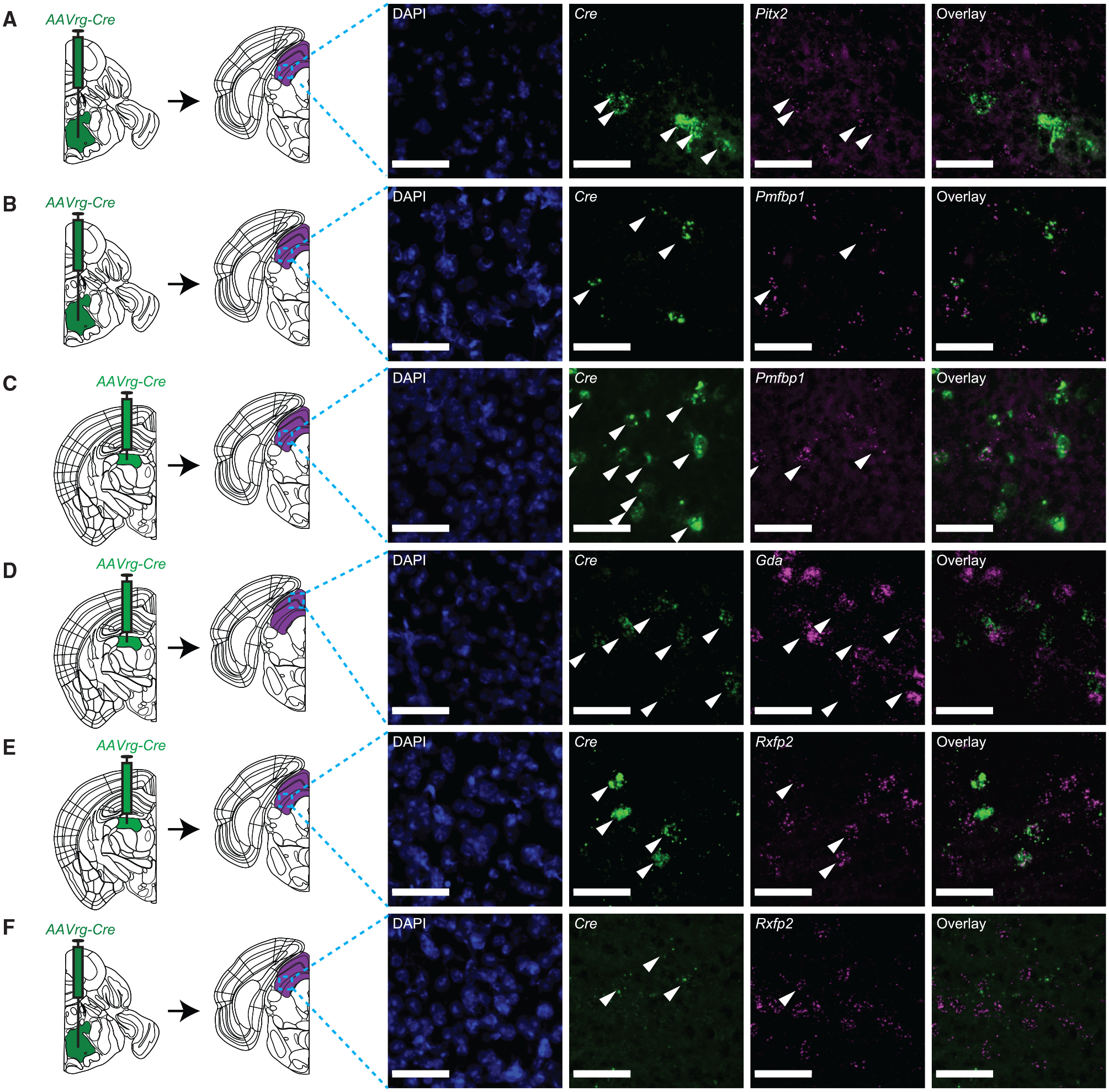
Analysis of candidate marker gene expression in different SC projection populations (A) Left, *AAVrg-Cre* was injected in right PPRF. Right, representative RNAscope images. Center left image shows the expression of *Cre*. Arrowheads indicate *Cre*^+^ cells. Center right image shows *Pitx2* expression. Arrowheads indicate *Cre*^+^ cells that are also *Pitx2*^+^. (B and C) As in (A), but for *Pmfbp1* expression in PPRF-projecting (B) and LP-projecting (C) neurons in deep SC. (D) As in (A)–(C) but for *Gda* expression in LP-projecting neurons in superficial SC. (E and F) As in (A)–(D) but for *Rxfp2* expression in LP-projecting (E) and PPRF-projecting (F) neurons in deep SC. Scale bars, 50 μm.

We used the Leiden algorithm to cluster cells (Figure 3B). Virtually all (98.54%, 53,740/54,537) of the nuclei were neuronal, indicating that the enrichment was successful. We subclustered excitatory neurons (60.8% of all neurons, 32,658/53,740), because most SC projection types are excitatory, identifying 25 clusters (Figures 3C–3E; inhibitory populations are shown in Figure S4C). Capitalizing on the laminar anatomy of SC, we analyzed the spatial distribution of markers with detectable expression in the Allen *in situ* hybridization database (Lein et al., 2007). Many localized to discrete laminae, while others localized to multiple laminae, consonant with findings in another recent study of SC (Figures S4A and S4B) (Xie et al., 2021). We then overlaid the expression of viral transgenes (Figures 3D and 3E). *GFP* reads were most prevalent in 2 clusters. Cluster 7 expressed *Pitx2*, a marker for deep SC neurons that drive orienting movements and project to contralateral PPRF and zona incerta (Masullo et al., 2019; Xie et al., 2021). Cluster 7 also expressed markers such as *Pmfbp1* with expression patterns in deep SC similar to that of *Pitx2*. To confirm that these markers were expressed in the PPRF-projecting population, we injected PPRF with *AAVrg-Cre* and SC with *AAV1-FLEX-tdTomato* (to provide signal amplification if *Cre* expression was too weak to detect *in situ*, which was not the case), waited 3 weeks, and used RNAscope FISH to determine whether *Cre*^+^ cells expressed *Pitx2* or *Pmfbp1* (Figure 4A) (Wang et al., 2012). Of 145 *Cre*^+^ cells, 120 (83%, n = 3 animals) also expressed *Pitx2* (Figure 4A). We next examined *Pmfbp1*. In the sequencing dataset, *Pmfbp1* was detected in a smaller fraction of the cells in this cluster than was *Pitx2*, suggesting that it is expressed at lower levels and leading us to predict it would be detected in fewer *Cre*^+^ cells. Consistent with this prediction, a smaller fraction of *Cre*^+^ cells was *Pmfbp1*^+^ (33/67, 49%, n = 4 animals) by FISH (Figure 4B). To confirm the specificity of *Pmfbp1* as a marker for this population, we examined its expression in a projection population that the sequencing dataset suggested expressed minimal amounts of *Pmfbp1*. We injected *AAVrg-Cre* into LP and used RNAscope to measure *Pmfbp1* co-expression in *Cre*^+^ cells (Figure 4C). Only 21 of 160 *Cre*^+^ cells were also *Pmfbp1*^+^ (13%, n = 5 animals), indicating that *Pmfbp1* is specific to PPRF-projecting cells (p < 0.0001, chi-square test) (Figure 4C). This result confirmed that *Pmfbp1* is expressed in deep SC neurons that express *Pitx2*. Thus, VECTORseq could identify a known subcortical projection population and additional markers for it, confirming the sensitivity and specificity of this approach.

We were surprised to detect *tdTomato* in virtually every neuronal population. This did not seem to be due to sequencing errors, because our reference library included control sequences that were not injected, such as *mCherry-IRES-Cre*, and that were not detected. To determine whether the retrograde labeling was promiscuous or had spilled over from CnF, which is near SC, we examined histology in the littermates of the cohort that was sequenced. SC contained sparse tdTomato-labeled fibers but no tdTomato-expressing cells, whereas GFP-labeled, PPRF-projecting cells were abundant (Figures S3C and S3D). This suggested that retrograde labeling was not promiscuous nor that there was spillover from the injection site. In contrast, the injection site was brightly labeled (Figure S3A). CnF abuts SC and was included in our microdissection, and we hypothesized that the abundant *tdTomato* reads were contaminated by ambient RNA released from CnF cells during dissociation, a common confound (Alvarez et al., 2020; Yang et al., 2020). When ambient RNA contamination is prevalent, highly expressed genes are found in many populations as the RNA becomes distributed throughout the nuclei suspension (Yang et al., 2020). Therefore, we predicted that *tdTomato* would be equally likely to be detected in neurons and non-neuronal cells. In contrast, if ubiquitous *tdTomato* detection in neuronal populations were due to retrograde infection rather than ambient RNA contamination, *tdTomato* should be found more frequently in neurons than in non-neuronal cells. Consistent with our hypothesis, 1.47% (797/54,267) of the total nuclei in our dataset and 1.34% (153/11,377) of the *tdTomato*^+^ nuclei were non-neuronal, suggesting that there was not a significant difference in the probability of detecting *tdTomato* in neuronal and non-neuronal cells (p = 0.34, chi-square test). In contrast, the other retrograde transgenes were significantly more likely to be detected in neurons: only 0.65% of *GFP*^+^ nuclei were non-neuronal (10/1,531, p = 0.009, chi-square test), and only 0.70% of *Cre*^+^ nuclei were non-neuronal (22/3117, p = 0.005, chi-square test). Thus, we conclude that the ubiquity of *tdTomato* reads is due to ambient RNA from extremely highly expressing cells in the injection site (CnF) that were included in the dissection. We did not further analyze *tdTomato*^+^ populations.

We then analyzed *HSV-Cre* labeling in SC excitatory neurons, observing that it was most prominent in two clusters, 10 and 11 (Figure 3E). Cluster 11 expressed markers such as *Tmem132c*, *Cbln2*, *Trhde*, and *Gda*, all of which localized to a thin lamina in the stratum opticum, where WF cells are found, in the Allen *in situ* dataset (Figure S4A); several, including *Gda* and *Cbln2*, were recently shown to be markers for WF cells (Xie et al., 2021) (Figure 3E). We used RNAscope to determine whether LP-projecting neurons in superficial SC express *Gda*, which appeared strongly expressed and specific within SC to the stratum opticum in the Allen *in situ* atlas (Figures 4D and S4A). Of 353 retrogradely labeled *Cre*^+^ cells in superficial SC, 307 (87%, n = 5 animals) also expressed *Gda*, confirming that it is a marker for WF cells (Figure 4D). We then analyzed the previously unknown LPMR-projecting population in intermediate and deep SC, cluster 10. This population expressed relatively few unique markers, including the specific but not highly expressed *Rxfp2*, which appeared to be expressed in deep SC in the Allen *in situ* atlas (Figures 3E and S4A). We used RNAscope to examine *Rxfp2* expression in the deep SC population that projects to LP. We injected *AAVrg-Cre* into LP, *AAV-CAG-FLEX-tdTomato* into SC (as noted previously, for signal amplification if needed), and waited 3 weeks before performing RNAscope (Figure 4E). *Rxfp2* was detected in 56 of 213 *Cre*^+^ cells (26%, n = 3 animals). We hypothesized this was reflective of low expression overall, because *Rxfp2*, although specific to cluster 10, was not highly expressed in the sequencing dataset (Figure 3E). Therefore, as a specificity control, we examined a population that the sequencing dataset suggested expressed minimal amounts of *Rxfp2*. We injected *AAVrg-Cre* into contralateral PPRF and used RNAscope to detect *Rxfp2* expression in *Cre*^+^ cells (Figure 4F). Only 1 of 29 *Cre*^+^ cells also expressed *Rxfp2* (3%, n = 4 animals), confirming the specificity of *Rxfp2* as a marker for the LPMR-projecting population in deep SC (p = 0.0065, chi-square test).

One potential concern is that viral infection could perturb endogenous gene expression and thereby not reveal the ground states of these projection populations. If that were the case, then we would expect that transgene^+^ cells would segregate from transgene^−^ cells within clusters. Importantly, transgene^+^ and transgene^−^ cells were interspersed in these clusters, indicating that endogenous gene expression is not skewed in cells infected with AAVrg or HSV and that these analyses reveal the cellular ground state (Figure S5). Thus, VECTORseq is compatible with multiple viral families, identifies both previously known and additional subcortical cell types, including a population that projects to LP, and reveals the cellular ground state.

### Application of VECTORseq to ventral midbrain inhibitory projection populations

**Figure 5.**
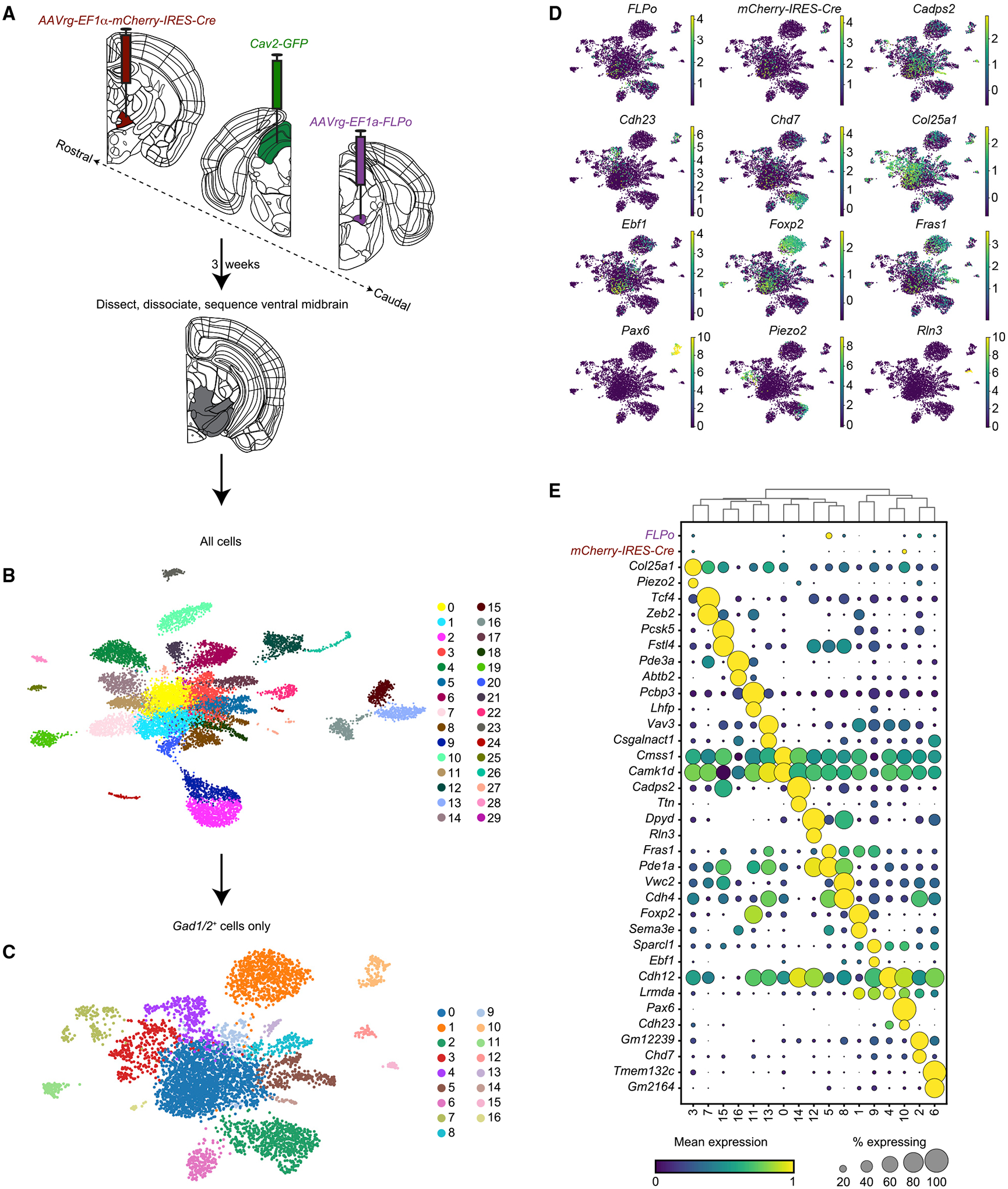
VECTORseq of ventral midbrain inhibitory neurons (A) Retrograde viruses were injected into right VM, right MLR, and left SC. Three weeks later, the right ventral midbrain was dissected; nuclei were isolated, stained, and sorted; and single-cell sequencing was performed. (B) UMAP plot of sequencing data and clustering. (C) UMAP plot of inhibitory (*Gad1*^+^*/Gad2*^+^) neurons. (D) UMAP plots of inhibitory neurons with viral transgene or example marker gene expression overlaid. (E) Enriched and differentially expressed genes in major ventral midbrain inhibitory cell types and distribution of virally encoded transgenes.

Many projection populations, especially in subcortical areas, are inhibitory. Therefore, we tested the applicability of VECTORseq to inhibitory populations. We focused on the ventral midbrain, where diverse inhibitory projection types in adjacent structures such as zona incerta (ZI) and substantia nigra pars reticulata (SNr), among others, innervate areas involved in movement control, such as ventromedial thalamus (VM), the mesencephalic locomotor region (MLR), and SC (Antal et al., 2014; Barthó et al., 2002; Hikosaka and Wurtz, 1983; McElvain et al., 2021; Nagalski et al., 2016; Watson et al., 2014). We injected VM and MLR with *AAVrg-mCherry-IRES-Cre* and *AAVrg-FLPo*, respectively (Figures 5A and S6). We had previously found that another widely used retrograde virus, canine adenovirus 2 (Cav-2), infected ventral midbrain neurons projecting to SC. Therefore, we injected contralateral SC with *Cav-2-GFP* (Figures 5A and S6) (Junyent and Kremer, 2015). In addition, we injected *AAV1-FLEX-tdTomato* in SNr as a fiducial for dissections (Figure 5A).

Three weeks later, we dissected the portion of the ventral midbrain containing ZI and SNr from 5 mice. Once again, we isolated *NeuN*^+^ nuclei, generated libraries using the 10x Chromium 5′ system, and performed Illumina paired-end sequencing using the NovaSeq platform. Analyses of the sequencing dataset revealed 34,274,388 reads corresponding to 13,412 cells. *FLP* and *Cre* were abundant in the sequencing dataset, at levels comparable to those of common marker genes (Table S3), whereas *Cav-2-GFP* was not detected. To investigate the lack of *GFP*, we analyzed identically injected mice histologically. Because Cav-2 infects both local neurons at the injection site and projections to that site, we examined the injection site in SC. This revealed only a few labeled cells (Figure S6C) that fell along the injection track. Thus, the lack of *GFP* reads in the sequencing dataset is likely due to a lack of infection.

**Figure 6.**
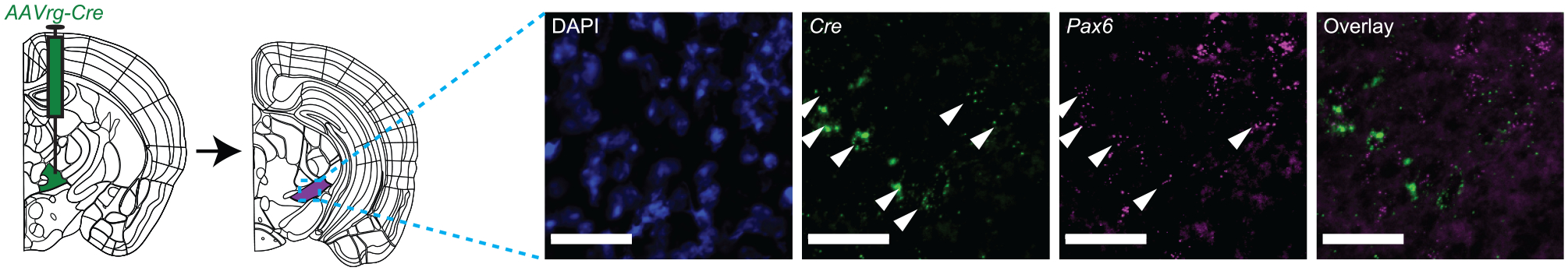
Analysis of candidate marker gene expression in ZI → VM population Left, *AAVrg-Cre* was injected in right VM. Three weeks later, mice were perfused and RNAscope was performed on ZI. Right, representative images. Center left image shows the expression of *Cre*. Arrowheads indicate *Cre*^+^ cells. Center right image shows *Pax6* expression. Arrowheads indicate *Cre*^+^ cells that are also *Pax6*^+^. Scale bars, 50 μm.

Once again, we clustered and annotated all cell types. Of 13,412 (91.81%) of the fluorescence-activated cell sorting (FACS)-sorted and profiled nuclei, 12,314 were neuronal (Figure 5B). We then separately subclustered excitatory and inhibitory neurons. This analysis yielded 7,019 excitatory (*Slc17a6*^+^) and 5,295 inhibitory (*Gad1*^+^*/Gad2*^+^) neurons (Figures 5C and S6D). We then overlaid viral transgene expression; because viral transgenes were most abundant in inhibitory subtypes, we focused subsequent analyses on inhibitory populations (Figures 5D, 5E, and S6D). *mCherry-IRES-Cre* and *FLPo* were present in several populations (Figure 5E). *mCherry-IRES-Cre* was notably abundant in a population that expressed *Pax6*, *Cdh23*, and *Pde11α* (Figure 5E). This was intriguing because *Pax6* and *Cdh23* have been reported to be expressed in ZI, particularly in the ventral subdivision in which the expression of GABAergic markers is dense, and the projection from ZI to VM has been shown to be GABAergic (Barthó et al., 2002; Watson et al., 2014). For this reason, we further pursued this population. We injected *AAVrg-Cre* into VM and used RNAscope to measure *Pax6* co-expression in *Cre*-expressing cells in ZI (Figure 6). Of 173 *Cre*^+^ cells, 123 (71%, n = 3 animals) expressed *Pax6*, confirming that it is a marker for this projection population. Thus, VECTORseq identified a subcortical inhibitory projection population.

To determine whether viral infection perturbed endogenous gene expression, we performed within-cluster comparisons of transgene^+^ and transgene^−^ cells. In 2 of the clusters, transgene^+^ cells were scattered throughout, suggesting that the viruses did not perturb endogenous gene expression, as we had observed in SC (Figures S7A–S7D). Interestingly, in the smallest population, the *Pax6*-expressing cluster 10, transgene^+^ cells were interspersed with transgene^−^ cells but concentrated in one region of the cluster (Figure S7E). After further subclustering, nearly all transgene^+^ cells fell into one of the subclusters, where they were interspersed with many transgene^−^ cells (Figure S7F). This suggested that cluster 10 may correspond to 2 closely related *Pax6*^+^ populations that were not distinguished during our initial clustering, perhaps due to the small number of cells or because they differed relatively subtly in gene expression. Upon further analysis, we identified a handful of markers that distinguish these 2 subpopulations, including *Gfra1*, *Unc13c*, *Cemip*, and *Ephb1* (Figures S7E–S7J). These analyses suggest that the retrograde viral transgenes did not perturb endogenous gene expression and that their distribution within clusters may be useful to guide subclustering of closely related subtypes differing in projection anatomy.

## DISCUSSION

The brain contains myriad projection neurons whose molecular and functional properties are unknown. One means to relate gene expression to projection anatomy is to start with gene expression by sequencing a structure, identifying markers for each cell type, and performing viral anterograde tracing of each using a panel of transgenic mice expressing Cre recombinase (Ding et al., 2020). This is a powerful, systematic approach, but it requires obtaining or generating and validating Cre transgenic mice for each population, and it is costly and mouse and labor intensive. For this reason, methods have been developed that start with anatomy, using retrograde labeling to selectively sequence projection neurons (Cadwell et al., 2016; Fuzik et al., 2016; Tasic et al., 2018). These approaches are fruitful but also fairly slow, laborious, costly, and involved, requiring specialized equipment and separate processing and sequencing for each population. In theory, different populations could be pooled for a single sequencing run to reduce costs, but attempting to pool samples with methods such as cell hashing, which are not widely used, would entail additional steps and costs, and can reduce both yield and data quality (Gaublomme et al., 2019; Stoeckius et al., 2018). Another recently described method, BARseq, requires costly specialized equipment to perform *in situ* sequencing, calibration of viral expression to avoid toxicity and perturbation of gene expression, and prior knowledge of markers for cell types in the tissue of interest (Chen et al., 2019). Therefore, we developed and validated an approach, VECTORseq, that enables a theoretically limitless number of projection populations to be barcoded simultaneously and identified without additional steps or specialized equipment. The isolated cells and nuclei can be sequenced in one run, rather than separate sequencing reactions for each projection target, reducing costs and increasing scalability. Thus, in comparison with existing approaches, VECTORseq is straightforward to implement and greatly reduces the number of animals sacrificed, sequencing costs, time, and steps (and potential failure points) required to characterize projection populations.

VECTORseq detected a variety of functionally different transgenes delivered by commonly used retrograde viruses such as AAVrg and HSV under the control of several promoters, including *Synapsin*, *CAG*, and *EF1α*; surprisingly, we also detected retrograde infection by AAV1, which is known to infect retrogradely but much less efficiently than AAVrg or HSV (Tervo et al., 2016). Thus, VECTORseq is a highly sensitive method that should be compatible with any viruses (e.g., Cav-2, lentivirus) that are used to target projection types (Wickersham and Feinberg, 2012). Importantly, comparison of infected and uninfected cells within clusters found no differences in expression of endogenous genes, and markers for virally labeled clusters were not enriched for inflammatory or antiviral genes, suggesting that both AAV and HSV did not perturb gene expression in these cell types. We combined as many as 6 different viruses and targeted up to 3 structures in individual proof-of-principle experiments, but future experiments could label a vast array of projection targets due to the diversity of available transgenes (e.g., recombinases, fluorescent proteins, optogenetic and chemogenetic tools) and the ability to distinguish closely related sequences such as *Cre* variants. For structures harboring many projection populations, such as SNr, which was recently shown to innervate 39 different targets (McElvain et al., 2021), it would be possible to increase labeling diversity by making custom AAVs containing noncoding barcode sequences.

We performed 5′ sequencing because many viruses share a 3′ UTR element (WPRE) that boosts transgene expression (Wang et al., 2016). Although most neuronal studies use 3′ sequencing, the core facilities we contacted offered 3′ and 5′ sequencing using commercial kits at identical costs and without additional steps for the end user. It may be possible to perform 3′ sequencing with VECTORseq by injecting viruses that lack the WPRE; however, one of our viruses, *hSyn-Dre*, lacked a WPRE, and only one read was detected for this virus, suggesting that the WPRE may increase RNA expression or stability and thus detectability (Wang et al., 2016). One alternative would be to include a gene-specific primer targeted to the 5’ end of WPRE to amplify the unique viral transgene sequences upstream of the WPRE. Another alternative would be to use methods that sequence through gene bodies; for example, we were able to detect viral transgenes using Smart-Seq2 (data not shown) (Picelli et al., 2014).

**Figure 7.**
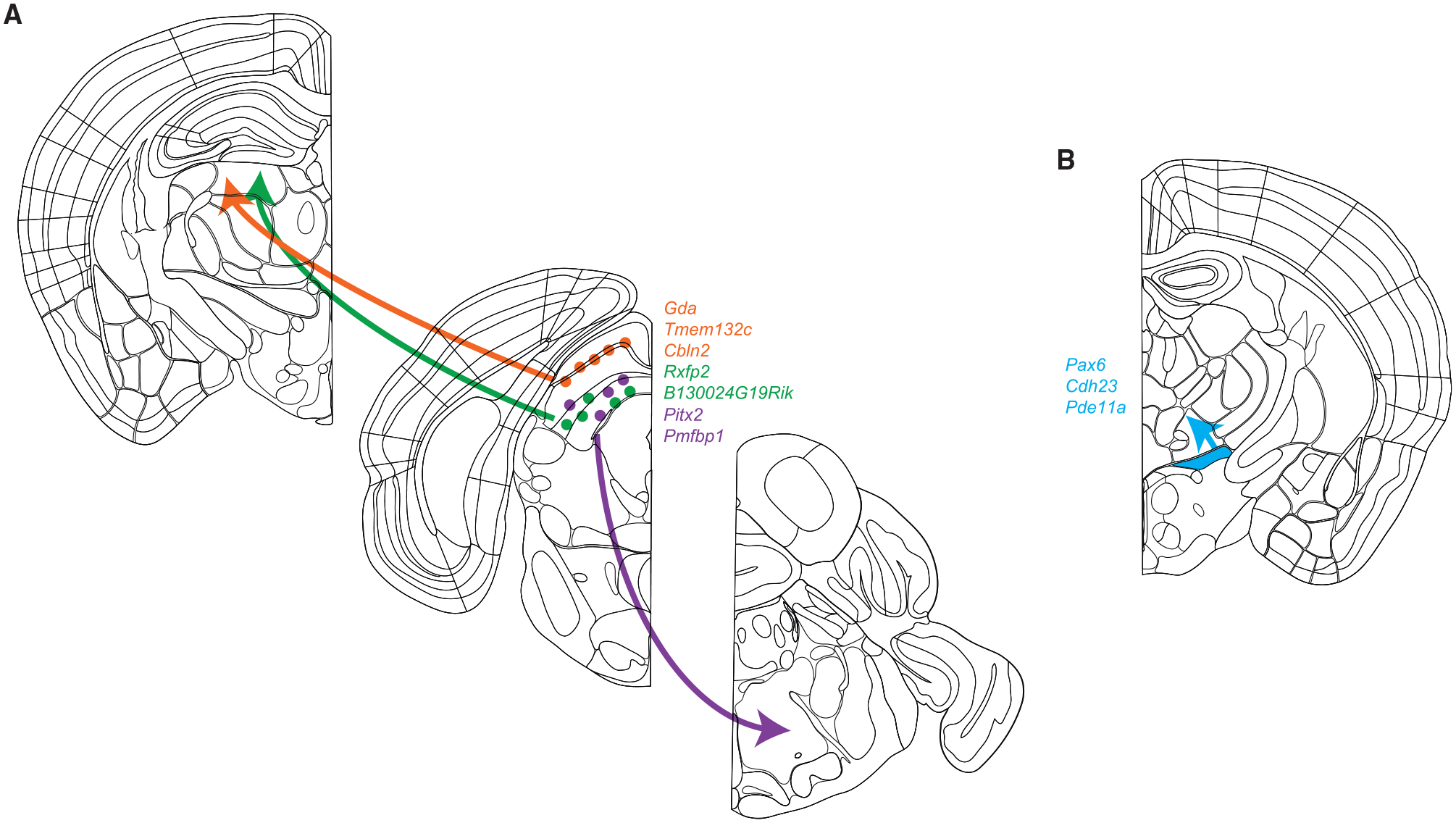
Summary of cell types and markers identified using VECTORseq (A) VECTORseq identified additional markers for SC WF cells (including *Gda*, *Tmem132c*, and *Cbln2*) that project to LPLR and for *Pitx2*^+^ cells that project to PPRF (*Pmfbp1*). In addition, VECTORseq identified a hitherto elusive population of deep SC neurons that project to LPMR labeled by *Rxfp2* and *B130024G19Rik*. (B) VECTORseq identified ZI *Pax6*^+^ cells as GABAergic neurons that project to thalamic nucleus VM.

We validated VECTORseq by labeling visual cortical projection populations, finding that viral barcodes were in excitatory neurons, as expected. We then combined validation and discovery by investigating structures with a mixture of known and unknown projections. First, VECTORseq identified a known SC projection population that innervates contralateral PPRF and expresses *Pitx2*, and uncovered an additional marker, *Pmfbp1* (Figure 7A) (Masullo et al., 2019; Xie et al., 2021). Second, we examined projections to thalamic nucleus LP, the rodent homolog of pulvinar. It is known that WF cells in superficial SC project to lateral LP, and a fortuitously identified Cre transgenic line (that does not recapitulate endogenous gene expression) has been widely adopted in the last few years for functional studies of WF cells and LP physiology (Gale and Murphy, 2014, 2016; Hoy et al., 2019; Reinhard et al., 2019; Sans-Dublanc et al., 2021). However, the molecular identity of WF cells was previously unknown. Using VECTORseq, we were able to define WF cells using SC transcriptional profiling (Figure 7A). Interestingly, a recent study used Patch-seq to transcriptionally profile this population, also finding that *Cbln2* and *Gda* are expressed in LP-projecting superficial SC neurons that appear to correspond to WF cells (Xie et al., 2021). Thus, our data match closely with those obtained using more labor-intensive and less-scalable approaches, providing further validation of VECTORseq. In addition to these known populations, we identified an elusive population. It was shown >45 years ago that the medial portion of pulvinar, the primate homolog of LPMR, receives input from the deep, oculomotor layers of SC (Benevento and Fallon, 1975). Anterograde tracing from mouse SC reveals similar projections to LPMR but the functional and molecular properties of this population are apparently unknown in any species (Gharaei et al., 2020). Using VECTORseq, we identified the deep SC population that projects to LPMR and markers for it, including *Rxfp2* and the highly expressed uncharacterized gene *B130024G19Rik*. Just as the discovery of a Cre transgenic line that labels WF cells has been transformative, these markers could enable functional studies of this elusive LP-projecting SC population and its role in sensory processing, sensorimotor integration, and behavior (Figure 7A). In this way, VECTORseq enabled both molecular characterization of defined populations and discovery of additional populations.

Finally, we performed VECTORseq on ventral midbrain neurons that project to several motor structures. This analysis defined a GABAergic population in ZI that expresses the marker *Pax6* and innervates VM (Figure 7B). Interestingly, a previous study found that GABAergic neurons in ZI project to VM, while a separate study analyzing gene expression suggested that *Pax6* was found in the ventral portion of ZI where GABAergic cells are enriched (Barthó et al., 2002; Watson et al., 2014). Here, we used an unbiased approach to link these disparate observations, illustrating the power of VECTORseq to solve correspondence problems and relate gene expression to connectivity and functional properties.

### Limitations of the study

Cav-2 was not detected in our ventral midbrain dataset. We believe that this is due to the batch of virus, because we had previously infected projections from ventral midbrain to SC using other batches of Cav-2. Nevertheless, viral tropism is an important consideration, and it is critical to confirm that a virus to be used in sequencing experiments infects the population of interest. As a potential solution, it may be desirable to heterologously express viral entry receptors, such as *hCar* for Cav-2, in the source structure of interest (Li et al., 2018). Conversely, our attempts to sequence projections to a structure very close to the source structure (SC to CnF) were confounded by ambient RNA from the injection site. Therefore, when examining projection targets near the source structure, it may be preferable to ensure that dissections exclude the injection site or to use a virus such as HSV, which is reported to express stably in retrogradely infected neurons but transiently at the injection site (Fenno et al., 2014). Relatedly, different cells within a source structure may project to adjacent targets, and it is important to ensure that viral infections into these targets are focal and avoid spillover. In such circumstances, it is advisable to perform confirmatory anterograde tracing using Cre transgenic mice and viral injections (Ding et al., 2020).

## STAR★METHODS

### RESOURCE AVAILABILITY

#### Lead contact

Requests for further information should be directed to and will be fulfilled by the Lead Contact, Evan Feinberg (evan.feinberg@ucsf.edu).

#### Materials availability

This study did not generate new unique reagents. Mice and all reagents used in this study are commercially available as indicated in the Key resources table.

#### Data and code availability

- The raw and processed single-cell sequencing data have been deposited at GEO: GSE189907 and are publicly available as of the date of publication.
- All code for analysis is publicly available at https://github.com/vic-cheung/vectorseq.
- Any additional information required to reanalyze the data reported in this paper is available from the lead contact upon request.

### EXPERIMENTAL MODEL AND SUBJECT DETAILS

#### Mouse breeding and husbandry

All experiments were performed according to Institutional Animal Care and Use Committee standard procedures. All mice were adult (8–12 weeks) male C57BL/6J. For each set of sequencing experiments, between 4–6 mice were used. For each set of RNAscope experiments, between 4–5 mice were used.

### METHOD DETAILS

#### Retrograde labeling

Mice were administered buprenorphine 30 minutes prior to anesthesia. 30 minutes later, mice were anesthetized with isoflurane and given meloxicam for analgesia. All coordinates are in mm. Angled injections were always done such that the tip of the syringe pointed toward the midline and the plunger tilted away from the midline. Injection coordinates were determined using an adult mouse atlas.

##### Injection coordinates and volumes (all measurements in mm)

**V1**, 35 nl/depth at a rate of 10 nl/minute at the coordinates:

1. AP: 2.69 posterior to bregma, ML: 2.50, DV: 0.60, 0.40 below pia
2. AP: 2.91 posterior to bregma, ML: 2.50, DV: 0.60, 0.40 below pia
3. AP: 3.15 posterior to bregma, ML: 2.50, DV: 0.75, 0.50 below pia
4. AP: 3.30 posterior to bregma, ML: 2.50, DV: 0.50, 0.25 below pia
5. AP: 3.51 posterior to bregma, ML: 2.50, DV: 0.50, 0.25 below pia
6. AP: 3.79 posterior to bregma, ML: 2.50, DV: 0.50, 0.25 below pia
7. AP: 4.00 posterior to bregma, ML: 2.50, DV: 0.50, 0.25 below pia
8. AP: 4.25 posterior to bregma, ML: 2.50, DV: 0.55, 0.40 below pia

**Striatum**, injection rate of 30nl/minute at the coordinates:

1. AP: 0.90 anterior to bregma, ML: 1.50, DV: 2.00 below pia, 150 nL at single depth
2. AP: 0.45 anterior to bregma, ML: 2.00 ML, DV: 2.00 below pia, 150 nL at single depth
3. AP: 0.00 at bregma, ML: 2.25, DV: 2.50 below pia, 150 nL at single depth
4. AP: 0.34 posterior to bregma, ML: 2.50, DV: 2.50 below pia, 150 nL at single depth

**SC**, 50 nl/depth at a rate of 30 nl/minute at the coordinates:

1. AP: 0.25 anterior to lambda, ML: 1.00, DV: 2.00, 1.75, 1.50, 1.25, 1.00 below skull surface
2. AP: 0.50 anterior to lambda, ML: 1.00, DV: 2.00, 1.75, 1.50, 1.25, 1.00 below skull surface

**Ventral midbrain**, 100 nl/depth at a rate of 30 nl/depth, 10° angle at the coordinates:

1. AP: 1.25 anterior to lambda, ML: 2.28, DV: 4.60, 4.40, 4.20 below skull surface

**VM**, injection rate of 30 nl/minute at the coordinates:

1. AP: 1.23 posterior to bregma, ML: 0.75, DV: 4.15 below skull surface, 150 nL at single depth
2. AP: 1.43 posterior to bregma, ML: 1.00, DV: 4.25 below skull surface, 50 nL at single depth
3. AP: 1.67 posterior to bregma, ML: 0.75, DV: 4.25 below skull surface, 50 nL at single depth

**MLR**, injection rate of 30 nl/minute at the coordinates:

1. AP: 4.23 posterior to bregma, ML: 1.30, DV: 3.83 below skull surface, 70 nL at single depth
2. AP: 4.43 posterior to bregma, ML: 1.25, DV: 3.63 below skull surface, 70 nL at single depth
3. AP: 4.63 posterior to bregma, ML: 1.20, DV: 3.80, 3.40 below skull surface, 30 nl/depth
4. AP: 4.83 posterior to bregma, ML: 1.50, DV: 3.50 below skull surface, 50 nL at single depth
5. AP: 4.89 posterior to bregma, ML: 1.00, DV: 3.00 below skull surface, 100 nL at single depth

**PPRF**, injection rate of 30 nl/minute at the coordinates:

1. AP: 4.95 posterior to bregma, ML: 0.63, DV: 5.13, 4.88, 4.63, and 4.38 below skull surface, 50 nL per depth
2. AP: 5.07 posterior to bregma, ML: 0.50, DV: 5.13, 4.88, 4.63, and 4.38 below skull surface, 50 nL per depth
3. AP: 5.19 posterior to bregma, ML: 0.50, DV: 5.13, 4.88, 4.63, and 4.50 below skull surface, 50 nL per depth
4. AP: 5.33 posterior to bregma, ML: 0.50, DV: 5.33, 5.25, 5.00, and 4.75 below skull surface, 50 nL per depth

**CnF**, 50 nl/depth at an injection rate of 30 nl/minute at the coordinates:

1. AP: 4.83 posterior to bregma, ML: 1.13, DV: 2.85 below skull surface
2. AP: 4.95 posterior to bregma, ML: 1.13, DV: 2.85 below skull surface
3. AP: 5.07 posterior to bregma, ML: 1.37, DV: 3.13, 2.85 below skull surface
4. AP: 5.19 posterior to bregma, ML: 1.25, DV: 2.85 below skull surface

**LP**, 70 nl/depth at an injection rate of 30 nl/minute at the coordinates:

1. AP: 1.55 posterior to bregma, ML: 1.00, DV: 2.63 below skull surface
2. AP: 1.67 posterior to bregma, ML: 1.00, DV: 2.63 below skull surface
3. AP: 1.79 posterior to bregma, ML: 1.50, DV: 2.63 below skull surface
4. AP: 1.91 posterior to bregma, ML: 1.37, DV: 2.63 below skull surface
5. AP: 2.03 posterior to bregma, ML: 1.30, DV: 2.50 below skull surface
6. AP: 2.15 posterior to bregma, ML: 1.25, DV: 2.60 below skull surface
7. AP: 2.27 posterior to bregma, ML: 1.37, DV: 2.60 below skull surface

#### Single-cell isolation

##### Tissue preparation

Mice were anesthetized and transcardially perfused with 4°C aCSF. Brains were quickly dissected out and placed in a chilled slurry of N-methyl-d-glucamine Buffer (NMDG Buffer). Brains were then glued in the coronal orientation onto a vibratome platform. The vibratome was filled with the cold NMDG buffer slurry. 300 μm sections were sliced at 0.06 mm/second and sections with the region of interest (ROI) were isolated. The slices were further micro-dissected to isolate the ROI. The ROI were recovered in a 37°C NMDG bath for 20 minutes before being placed in room temperature aCSF for 20 minutes.

#### Single-cell dissociation

Tissue was processed using the Papain Dissociation System Protocol (Worthington Biochemical Corporation, LK003150). In summary, this protocol involved gentle trituration using a transfer pipette (Falcon, 357524) every 20 minutes for 1–1.5 hours using the provided dissociation buffers in a 37°C rocker. After dissociation, the suspension was centrifuged in a low-bind microcentrifuge tube (Eppendorf) at 300 *g* for 10 minutes. After centrifugation and removal of supernatant, the pellet was resuspended with provided albumin-ovomucoid inhibitor. Cell debris was removed using the provided density gradient solutions, spinning in a centrifuge at 100 *g* for 7 minutes. Supernatant was discarded; cell pellet was reconstituted in 1mL of aCSF. Cells were counted using a hemocytometer and diluted or concentrated to roughly 450 cells/μL. 10x Genomics 5′ v1.1 library prep and NextSeq sequencing were performed by the Gladstone Institute Genomics core.

#### Single nuclei isolation

##### Tissue preparation

Mice were anesthetized and trans-cardially perfused with 4°C aCSF. Brains were quickly dissected out and placed in clean 4°C aCSF. Brains were then glued in the coronal orientation onto a vibratome platform. The vibratome was filled with 4°C aCSF. 300 μm sections were cut at 0.06 mm/second and sections containing the ROI were isolated. The ROI was micro-dissected out of the brain slices, diced into rice-sized pieces, and then placed in a low-bind microcentrifuge tube (Eppendorf). The tissue was then flash-frozen using liquid nitrogen.

#### Single nuclei dissociation

We followed a published protocol (Martin et al., 2020). Briefly, all steps were done either in 4°C or on ice. All reagents and items used were pre-chilled overnight at 4°C. Flash frozen tissue was gently triturated with detergent-based extraction buffer until tissue was visibly broken up, careful not to generate any bubbles. The entire volume was then passed through a 26G needle twice before transfer into a pre-chilled 50mL Falcon tube. 30 mL of wash buffer (HEPES-based buffer with 10% BSA) was added. This volume was then split into 2 15 mL centrifuge tubes for centrifugation at 600 g for 10 minutes at 4°C. Supernatant was removed until roughly 500 μL remained in each tube. Samples were then pooled together (total volume = 1 mL). The suspension was then passed through a pre-chilled 40 μm cell strainer and filtered using only gravity. Nuclei were counted using a hemocytometer and diluted or concentrated to roughly 8–10 million nuclei/mL. 200 μL of nuclei were reserved as a negative control. The remaining volume of nuclei was stained with rabbit anti-NeuN antibody conjugated to AlexaFluor488 (Abcam, ab190195) at a concentration of 0.1–10 μg/mL for 30 minutes in the dark on a gentle rocker. Stained nuclei were then washed with FACS buffer, centrifuged at 200 *g* for 1 minute, and supernatant was aspirated. The pellet was then resuspended in 1 mL of FACS buffer. DAPI was then added at 1 μg/μL. FACS was performed at the Gladstone Institute Flow Core on an Aria II. 10x Genomics 5′ v1.1 and v2 library prep was performed by the Gladstone Institute Genomics core. Sequencing of the library was done with UCSF’s CAT core.

#### Sequencing analysis, QC, and clustering

Analyses were performed in Python. 10x Genomics’ Cellranger cli was used to add viral transgenes to their mouse (mm10) reference genome (Zheng et al., 2017). Fastq outputs were aligned to this customized mm10 reference genome; introns were included in the alignment. Outputs of the alignments include a count matrix. Using Scanpy, cells with more than 5% mitochondrial gene expression were excluded. For each dataset, the distribution of genes and counts per cell were plotted and lower bound and upper bound cutoffs were chosen based on these distributions (Luecken and Theis, 2019). For the V1 dataset, cells with fewer than 200 genes or 750 counts or more than 6,000 genes or 30,000 counts were excluded. For the SC dataset cells with fewer than 300 genes or 750 counts or more than 9,000 genes or 50,000 counts were excluded. For the ventral midbrain dataset, cells with fewer than 300 genes or 500 counts or more than 5,000 genes or 20,000 counts were excluded. Doublets were removed using Scrublet (Wolock et al., 2019). Counts in each cell were then normalized to 10^4^ and then log-normalized (Wolf et al., 2018). We implemented a version of term frequency-inverse document frequency (TF-IDF) normalization using the formula:

TF=Term Frequency (number of reads)

N = total number of cells

n = number of cells in which the gene appears

TF-IDF is useful for weighting genes according to their variance across the population rather than their absolute expression (Moussa and Măndoiu, 2018). After normalization steps, the top 2000 highly variable genes were selected. Data were subset using these highly variable genes. The subset data were then scaled to unit variance and zero-centered. Data preprocessing in Scanpy involved dimensionality reduction using principal component analysis (PCA) with 50 principle components and *svd_solver* set to “*ar-pack*,” followed by constructing a neighborhood graph using 15 nearest neighbors. Only endogenous genes were used to create the neighborhood graph to avoid any potential influence of viral transgenes on clustering. The Leiden algorithm was applied to the neighborhood graph with resolution 0.6 to generate clusters. Uniform manifold approximation projection (UMAP) was applied to the neighborhood graph to visualize the resultant clusters in 2 dimensions. Dendrogram plots were generated using complete-linkage hierarchical clustering using Pearson’s correlation coefficient and top 50 principal components.

#### Marker gene selection

Top 50 genes from each cluster were selected using the Mann-Whitney *U* test with Benjamini-Hochberg procedure to control for false discovery rate. Gene expression heatmaps were generated of the top 50 differentially expressed genes from each cluster. Clusters were merged based on visual analysis of heatmaps, dendrogram plots, and applying an 80% cut-off to mutual presence of the top 50 unique genes across cluster pairs using the Jaccard similarity score. After cluster merging, genes that are unique to a specific cluster, highly expressed, and expressed in the majority of that specific cluster were selected as marker genes. Subsets of these unique genes were selected as biomarkers of interest for *in situ* hybridization using RNAscope.

#### Histology

##### Tissue preparation for native fluorescence

Mice were anesthetized with 100% isoflurane and transcardially perfused first with dPBS and subsequently with 10% formalin solution for fixation. Brains were then harvested and post-fixed in 10% formalin for 4–12 hours at 4°C. After post-fixation, brains were transferred to a 20% sucrose solution and kept at 4°C until the tissue was saturated with sucrose and no longer floating in solution. Brains were then frozen in OCT (Sakura) and sectioned coronally via cryostat at a thickness of 50 μm and imaged using a Zeiss LSM 700 laser scanning confocal microscope.

##### Tissue preparation for in situ hybridization

Mice were rapidly anesthetized with 100% isoflurane and decapitated. Brains were quickly dissected and placed in OCT-filled cryosectioning cubes and immediately transferred into a slurry bath of 100% ethanol and dry ice for flash freezing. Frozen brains were stored at −80°C until ready for use. Brains were cryo-sectioned at −16°C. Each section was 15 μm thick. Each section was directly mounted onto *Superfrost Plus* slides (Fisher) and dried for at least 30 minutes inside the cryostat chamber before storage at −80°C.

#### RNAscope

All experiments were performed according to the Advanced Cell Diagnostics (ACD) RNAscope protocol. Each Target Probe contains a mixture designed to bind to a specific target RNA. Each of these probes were detectable in one of three color channels, C1, C2, and C3 as follows: C1, Alexa 488 nm; C2, Atto 550 nm; C3, Atto 647 nm. In each experiment, we probed for *Cre* in channel 1(C1), *tdTomato* in channel 2 (C2), and the gene of interest (*Gda, Pax6, Pitx2, Pmfbp1, Rxfp2*) in channel 3 (C3). Briefly, tissue was immediately fixed at 4°C in pre-chilled formalin for 15 minutes following removal from storage at −80°C. All dehydration and wash steps were performed at room temperature using 50% ethanol, 70% ethanol, and 100% ethanol. Protease IV pretreat (from the RNAscope Fluorescent Assay v1 kit) was used to permeabilize the tissue. Incubation occurred at room temperature for no more than 30 minutes to prevent over-digestion. After pretreatment incubation, slides were washed twice in PBS with gentle agitation for 30 s. The probe mix was applied to each slide and incubated for 2 hours at 40°C. After probe incubation, slides were washed twice for 2 minutes each in RNAscope Buffer at room temperature. Probe hybridization signals were augmented using sequential hybridization of 4 amplifiers. Incubation times varied by amplification step but were all performed at 40°C. Between each amplification incubation step, slides were washed twice for 2 minutes each in RNAscope buffer at room temperature. Amp4 Alt B-FL was used for the last amplification step. After the last wash, slides were coverslipped and imaged. RNAscope images in figures are pseudocolored for accessibility.

### QUANTIFICATION AND STATISTICAL ANALYSIS

Statistical methods were not used to predetermine sample sizes. Sequencing data collection was not randomized or blinded because there was a single experimental condition for each dataset. RNAscope data were collected and scored blindly.

## Supplementary Material

1

2

## Figures and Tables

**Table:** KEY RESOURCES TABLE

REAGENT or RESOURCE	SOURCE	IDENTIFIER
Antibodies		
Rabbit anti-NeuN, conjugated AlexaFluor 488	Abcam	Cat# ab190195; RRID:AB_2716282
Bacterial and virus strains		
*AAVrg-CAG-GFP*	Addgene	Cat# 37825-AAVrg; RRID:Addgene_37825
*AAVrg-CAG-tdTomato*	Addgene	Cat# 59462-AAVrg; RRID:Addgene_59462
*AAVrg-Ef1α-mCherry-IRES-Cre*	Addgene	Cat# 55632-AAVrg; RRID:Addgene_55632
*AAVrg-Ef1α-FLPo*	Addgene	Cat# 55637-AAVrg; RRID:Addgene_55637
*AAVrg-hSyn-Dre*	Addgene	Cat# 50363-AAVrg; RRID:Addgene_50363
*HSV-hEF1α-Cre*	MGH Gene Delivery Technology Core	Cat# RN425
*Cav-2-GFP*	IGMM	N/A
*AAVrg-hSyn-Cre*	Addgene	Cat# 105553-AAVrg; RRID:Addgene_105553
*AAV1-CAG-FLEX-tdTomato*	Addgene	Cat# 28306-AAV1; RRID:Addgene_28306
Critical commercial assays		
Papain Dissociation System Protocol	Worthington Biochemical Corporation	Cat# LK003150
5′ v2 Library prep kit	10x Genomics	Cat# 1000265
Chromium Next GEM Chip K Single Cell Kit, 16 rxns	10x Genomics	Cat# 1000287
Chromium Next GEM Single Cell 5′ Library and Gel Bead Kit v1.1, 4 rxns	10x Genomics	Cat# 1000167
Chromium Next GEM Chip G Single Cell Kit, 16 rxns	10x Genomics	Cat# 1000127
Neuron isolation kit	Miltenyi	Cat# 130-126-603
RNAscope Fluorescent Multiplex Detection Reagents	Advanced Cell Diagnostics	Cat# 320851
RNAscope Probe - Mm-Gda-C3	Advanced Cell Diagnostics	Cat# 520531-C3
RNAscope Probe - Mm-Pmfbp1-C3	Advanced Cell Diagnostics	Cat# 504111-C3
RNAscope Probe - Mm-Rxfp2-C3	Advanced Cell Diagnostics	Cat# 589261-C3
RNAscope Probe - Mm-Pitx2-C3	Advanced Cell Diagnostics	Cat# 412841-C3
RNAscope Probe - Mm-Pax6-C3	Advanced Cell Diagnostics	Cat# 412821-C3
RNAscope Probe - CRE-C1	Advanced Cell Diagnostics	Cat# 312281-C1
Deposited data		
Raw data	GEO: GSE189907	N/A
Analysis	https://github.com/vic-cheung/vectorseq	N/A
Experimental models: Organisms/strains		
C57BL/6J *Mus musculus*	The Jackson Laboratory	Cat# 000664; RRID:IMSR_JAX:000664
Software and algorithms		
Cellranger 6.0.0	Zheng et al., 2017	https://10xgenomics.com/; RRID:SCR_017344
Scanpy 1.7.2	Wolf et al., 2018	https://github.com/theislab/scanpy; RRID:SCR 018139
Scrublet 0.2.1	Wolock et al., 2019	https://github.com/swolock/scrublet; RRID:SCR_018098
Python 3.9	N/A	https://www.python.org/downloads/; RRID:SCR_008394
Other		
Transfer pipette	Falcon	Cat# 357524
Microcentrifuge tube (Low-bind)	Eppendorf	Cat# 02681321
Zeiss LSM 700 laser scanning confocal microscope	N/A	N/A
AriaII FACS Sorter	N/A	N/A; RRID:SCR_018091
Superfrost Plus slides	Fisher	Cat #1255015

## Highlights

- Retrograde viruses express mRNA at levels detectable in single-cell sequencing
- Different transgenes can be multiplexed in a single sequencing run
- VECTORseq identifies both cortical and subcortical projection neurons
- VECTORseq defines new superior colliculus and zona incerta projection populations
