# Supplementary material for: Virally encoded connectivity transgenic overlay RNA sequencing (VECTORseq) defines projection neurons involved in sensorimotor integration
Source: Cell Rep. Author manuscript; Available in PMC 2021 Dec 31. (PMC8719358; doi:10.1016/j.celrep.2021.110131)
Supplement: 1 [file NIHMS1766437-supplement-1.pdf]

Cell Reports, Volume *37*

Supplemental information

Virally encoded connectivity transgenic overlay RNA sequencing (VECTORseq) defines projection neurons involved in sensorimotor integration

Victoria Cheung, Philip Chung, Max Bjorni, Varvara A. Shvareva, Yesenia C. Lopez, and Evan H. Feinberg

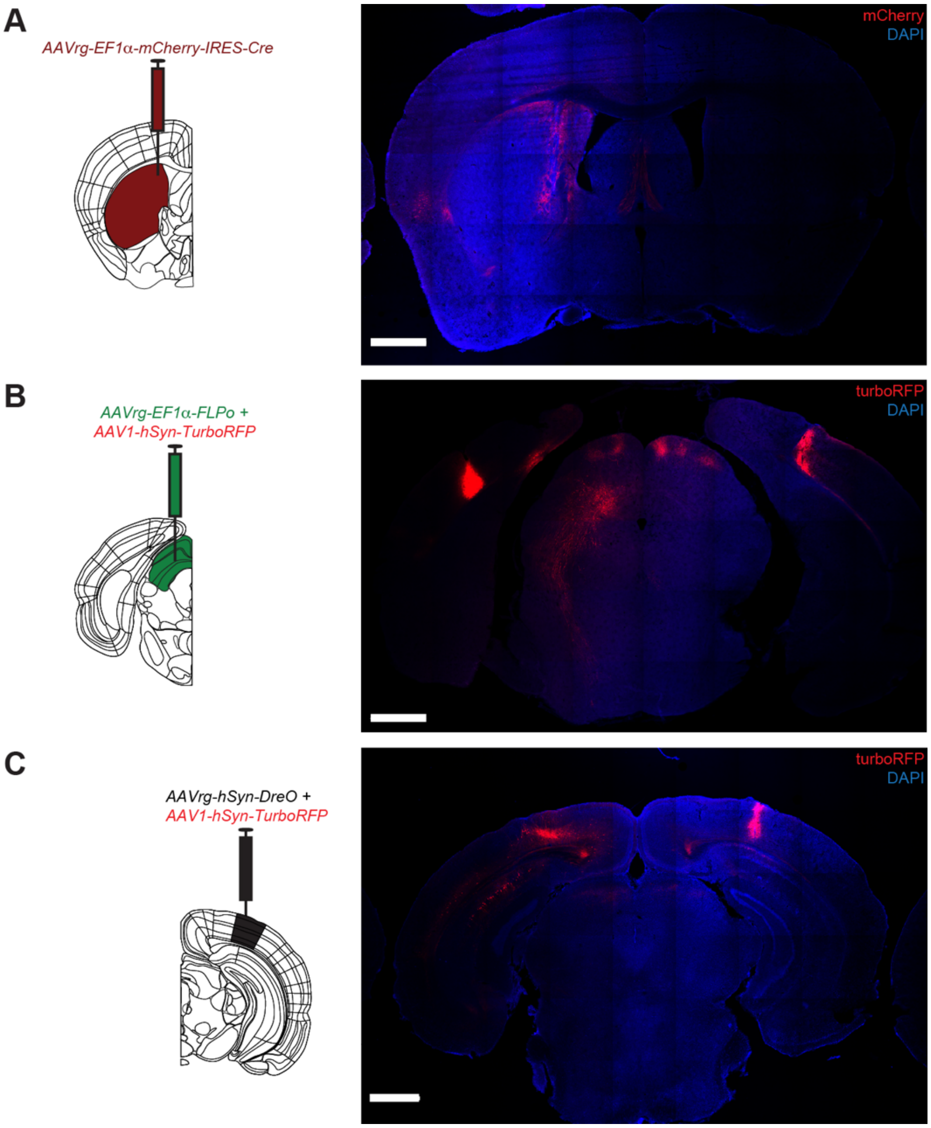

Figure S1. Injection sites for V1 retrograde labeling experiment. Related to Figure 2. (A) Injection site in left striatum. (B) Injection site in left SC. Labeling is also visible in injection site in right V1 (see C) and retrograde labeling is visible in left V1. (C) Injection site in right V1. Retrograde labeling is also visible in left V1. Scale bars, 1 mm.

```
mCherry-IRES-Cre  ---------------------ATGTCCAATTTACTGACCGTACACCAAAATTTGCCTGCATTACCGGTCGATGCAACGAG
HSV-Cre           ATGCCCAAGAAGAAGAGGAAGGTGTCCAATCTCCTGACTGTTCACCAGAACCTCCCTGCGCTGCCAGTAGATGCCACTAG
                                        ******** * ***** ** ***** **  * *****  * ** ** ***** ** **

mCherry-IRES-Cre  TGATGAGGTTCGCAAGAACCTGATGGACATGTTCAGGGATCGCCAGGCGTTTTCTGAGCATACCTGGAAAATGCTTCTGT
HSV-Cre           CGATGAGGTCAGGAAAAATCTCATGGATATGTTTAGGGATAGACAGGCGTTTTCTGAACACACCTGGAAAATGCTGCTTA
                   ********  * ** ** ** ***** ***** ****** * ************** ** ************** **

mCherry-IRES-Cre  CCGTTTGCCGGTCGTGGGCGGCATGGTGCAAGTTGAATAACCGGAAATGGTTTCCCGCAGAACCTGAAGATGTTCGCGAT
HSV-Cre           GCGTGTGCCGATCCTGGGCAGCCTGGTGTAAGCTGAACAATCGCAAATGGTTCCCCGCCGAGCCGGAGGACGTGCGCGAT
                   *** ***** ** ***** ** ***** *** **** ** ** ******** ***** ** ** ** ** ** ******

mCherry-IRES-Cre  TATCTTCTATATCTTCAGGCGCGCGGTCTGGCAGTAAAAACTATCCAGCAACATTTGGGCCAGCTAAACATGCTTCATCG
HSV-Cre           TACCTGCTGTATCTCCAGGCAAGAGGGCTGGCTGTCAAGACTATCCAGCAGCACTTGGGCCAACTGAATATGCTGCATCG
                  ** ** ** ***** *****  * ** ***** ** ** *********** ** ******** ** ** ***** *****

mCherry-IRES-Cre  TCGGTCCGGGCTGCCACGACCAAGTGACAGCAATGCTGTTTCACTGGTTATGCGGCGGATCCGAAAAGAAAACGTTGATG
HSV-Cre           ACGCAGCGGGCTCCCCCGGCCTAGCGATTCAAACGCAGTCTCCCTTGTTATGAGGAGAATTAGAAAGGAAAACGTAGATG
                   **   ****** ** ** ** ** **    ** ** ** ** ** ****** ** * **  **** ******** ****

mCherry-IRES-Cre  CCGGTGAACGTGCAAAACAGGCTCTAGCGTTCGAACGCACTGATTTCGACCAGGTTCGTTCACTCATGGAAAATAGCGAT
HSV-Cre           CGGGTGAGAGGGCTAAGCAGGCTCTCGCTTTTGAGCGGACTGATTTCGACCAGGTCAGATCCCTGATGGAGAACAGCGAT
                  * *****  * ** ** ******** ** ** ** ** *****************  * ** ** ***** ** ******

mCherry-IRES-Cre  CGCTGCCAGGATATACGTAATCTGGCATTTCTGGGGATTGCTTATAACACCCTGTTACGTATAGCCGAAATTGCCAGGAT
HSV-Cre           CGGTGCCAGGACATCAGGAACCTCGCATTTCTGGGAATTGCATATAACACACTTCTGCGCATAGCTGAGATCGCCCGGAT
                  ** ******** **  * ** ** *********** ***** ******** **  * ** ***** ** ** *** ****

mCherry-IRES-Cre  CAGGGTTAAAGATATCTCACGTACTGACGGTGGGAGAATGTTAATCCATATTGGCAGAACGAAAACGCTGGTTAGCACCG
HSV-Cre           CAGAGTGAAAGACATCAGTCGAACGGACGGCGGCCGGATGCTTATTCATATTGGACGCACAAAGACATTGGTCAGCACCG
                  *** ** ***** ***   ** ** ***** **  * *** * ** ********  * ** ** **  **** *******

mCherry-IRES-Cre  CAGGTGTAGAGAAGGCACTTAGCCTGGGGGTAACTAAACTGGTCGAGCGATGGATTTCCGTCTCTGGTGTAGCTGATGAT
HSV-Cre           CTGGCGTTGAAAAGGCCTTGTCCCTGGGCGTAACGAAGCTGGTGGAAAGATGGATCTCAGTGTCCGGCGTGGCTGACGAC
                  * ** ** ** *****  *   ****** ***** ** ***** **  ******* ** ** ** ** ** ***** **

mCherry-IRES-Cre  CCGAATAACTACCTGTTTTGCCGGGTCAGAAAAAATGGTGTTGCCGCGCCATCTGCCACCAGCCAGCTATCAACTCGCGC
HSV-Cre           CCTAATAATTACTTGTTCTGTCGAGTGAGAAAAAACGGAGTCGCCGCGCCCTCTGCCACCAGCCAATTGAGTACACGGGC
                  ** ***** *** **** ** ** ** ******** ** ** ******** **************  *    ** ** **

mCherry-IRES-Cre  CCTGGAAGGGATTTTTGAAGCAACTCATCGATTGATTTACGGCGCTAAGGATGACTCTGGTCAGAGATACCTGGCCTGGT
HSV-Cre           CCTTGAAGGGATCTTTGAGGCAACCCACCGACTCATATACGGAGCCAAGGATGACAGTGGCCAGAGGTATCTCGCCTGGT
                  *** ******** ***** ***** ** *** * ** ***** ** *********  *** ***** ** ** *******

mCherry-IRES-Cre  CTGGACACAGTGCCCGTGTCGGAGCCGCGCGAGATATGGCCCGCGCTGGAGTTTCAATACCGGAGATCATGCAAGCTGGT
HSV-Cre           CAGGTCATTCTGCTAGGGTGGGGGCCGCACGAGACATGGCGCGGGCAGGAGTCTCCATACCAGAGATTATGCAAGCTGGA
                  * ** **   ***  * ** ** ***** ***** ***** ** ** ***** ** ***** ***** ***********

mCherry-IRES-Cre  GGCTGGACCAATGTAAATATTGTCATGAACTATATCCGTAACCTGGATAGTGAAACAGGGGCAATGGTGCGCCTGCTGGA
HSV-Cre           GGTTGGACAAATGTGAACATCGTTATGAACTATATCCGCAATCTTGACTCTGAAACCGGGGCCATGGTGAGACTGCTCGA
                  ** ***** ***** ** ** ** ************** ** ** **   ****** ***** ****** * ***** **

mCherry-IRES-Cre  AGATGGCGATTAG
HSV-Cre           AGATGGTGACTGA
                  ****** ** *
```

Figure S2: Alignment of *Cre* coding sequences used in the study. Related to Figure 2 and Table S1. Alignments were performed using ClustalW. Asterisks indicate identities and gaps indicate mismatches. The *Cre* sequence in *mCherry-IRES-Cre* contains a 5’ SV40 nuclear localization signal.

Transgenes	Number of expressing cells	Number of reads	Abundance in expressing cells
Dre	1	1	0.007
FLPo	216	8,007	0.43 +/- 1.38
mCherry-IRES-Cre	3	15	0.70 +/- 0.05
turboRFP	35	114	0.05 +/- 0.06
EYFP	350	10,045	0.85 +/- 2.73
tdTomato	769	33,982	0.96 +/- 3.38
Endogenous Genes			
Snap25	313	3,522	0.14 +/- 0.14
Rbfox3	138	459	0.04 +/- 0.03
Slc17a6	72	152	0.04 +/- 0.03
Camk2a	169	912	0.07 +/- 0.06
Gad1	44	265	0.04 +/- 0.05
Gad2	10	176	0.07 +/- 0.04
Mog	1,482	19,362	0.21 +/- 0.12
Flt1	1,654	20,198	0.27 +/- 0.21

Table S1: Transgene detection in V1 sequencing dataset. Related to Figure 2. First column lists transgenes and endogenous genes for comparison. Second column indicates the number of cells in which each transgene or endogenous gene was detected. Third column indicates total number of reads corresponding to transgenes or endogenous genes in V1 sequencing dataset overall. Fourth column indicates relative expression of transgenes and common marker genes. To control for differences in abundance of different cell types, values denote the mean percentage of reads +/- standard deviation corresponding to a given marker or transgene in cells positive for that marker or transgene.

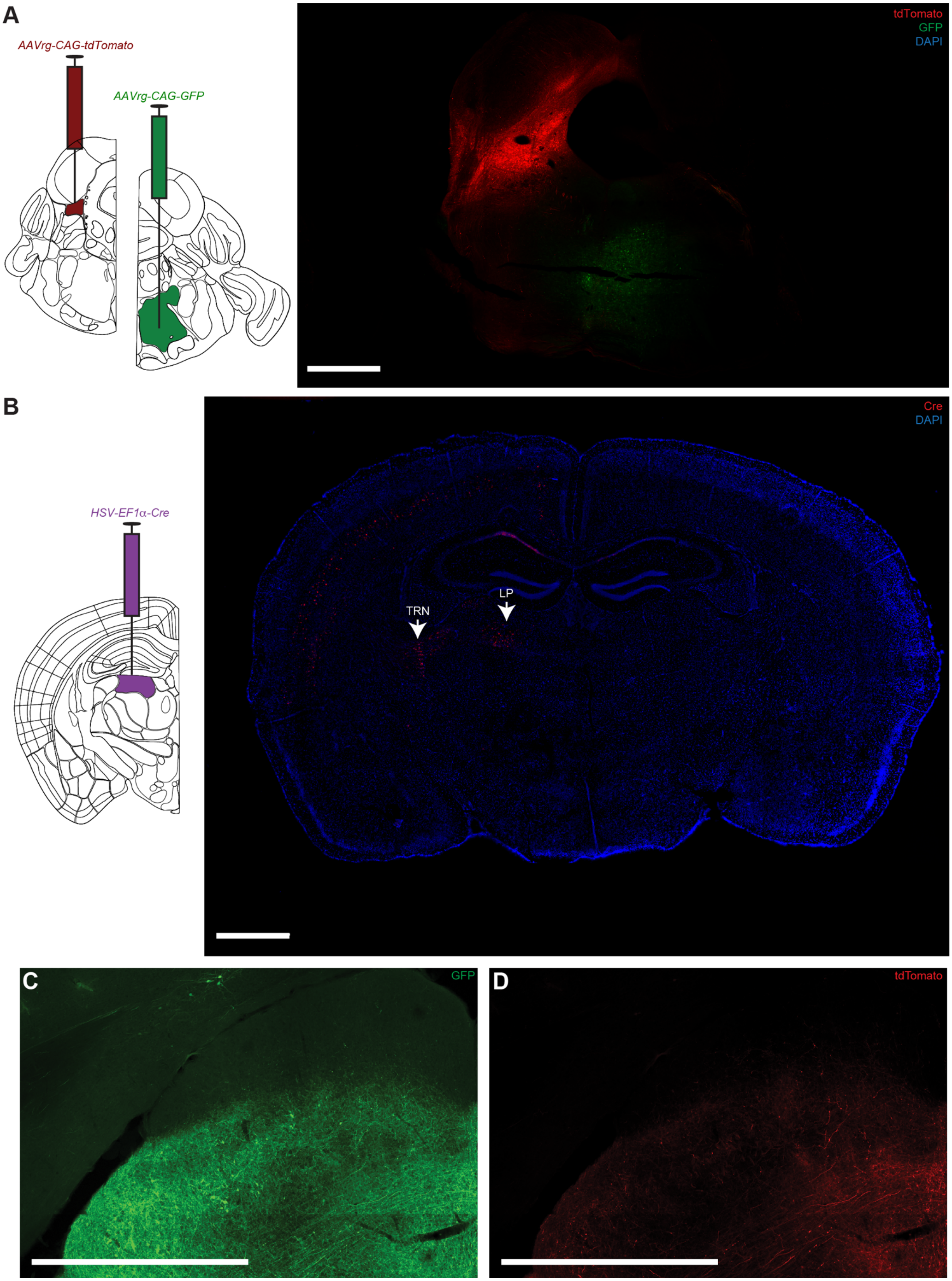

A
AAVrg-CAG-tdTomato
AAVrg-CAG-GFP
tdTomato
GFP
DAPI
B
HSV-EF1α-Cre
Cre
DAPI
TRN
LP
C
GFP
D
tdTomato

Figure S3. Injection site histology for SC experiment of retrograde labeling from CnF in SC. Related to Figure 3. (A) Native fluorescence in injection sites in CnF and PPRF. (B) Injection site in LP. Because Cre is not intrinsically fluorescent, RNAscope was used to visualize its expression. Retrograde labeling is visible in structures that project to LP, including thalamic reticular nucleus (TRN) and layer 5 of overlying cortex (Mease and Gonzalez, 2021; Wang et al., 2001). (C) Retrogradely infected SC neurons from *AAVrg-CAG-GFP* injection into contralateral PPRF. (D) Only sparse $tdTomato^+$ fibers and no $tdTomato^+$ retrogradely labeled cell bodies are seen in SC from injection of *AAVrg-CAG-tdTomato* in left CnF. Scale bars, 1 mm.

Transgenes	Number of expressing cells	Number of reads	Abundance in expressing cells
Cre	3,117	15,861	0.05 +/- 0.13
GFP	1,531	5,270	0.03 +/- 0.04
tdTomato	11,377	170,025	0.70 +/- 0.05
Endogenous Genes			
Snap25	53,717	617,837	0.10 +/- 0.05
Rbfox3	45,279	155,127	0.03 +/- 0.02
Slc17a6	31,559	126,995	0.03 +/- 0.02
Camk2a	39,291	121,268	0.03 +/- 0.03
Gad1	26,957	116,424	0.04 +/- 0.04
Gad2	28,342	159,007	0.05 +/- 0.04
Mog	2,842	4,856	0.02 +/- 0.04
Flt1	86	469	0.07 +/- 0.13

Table S2. Transgene detection in SC sequencing dataset. Related to Figure 3. First column lists transgenes and endogenous genes for comparison. Second column indicates the number of cells in which each transgene or endogenous gene was detected. Third column indicates total number of reads corresponding to transgenes or endogenous genes in SC sequencing dataset overall. Fourth column indicates relative expression of transgenes and common marker genes. As a control for differences in abundance of different cell types, values denote the mean percentage of reads +/- standard deviation corresponding to a given marker or transgene in cells positive for that marker or transgene.

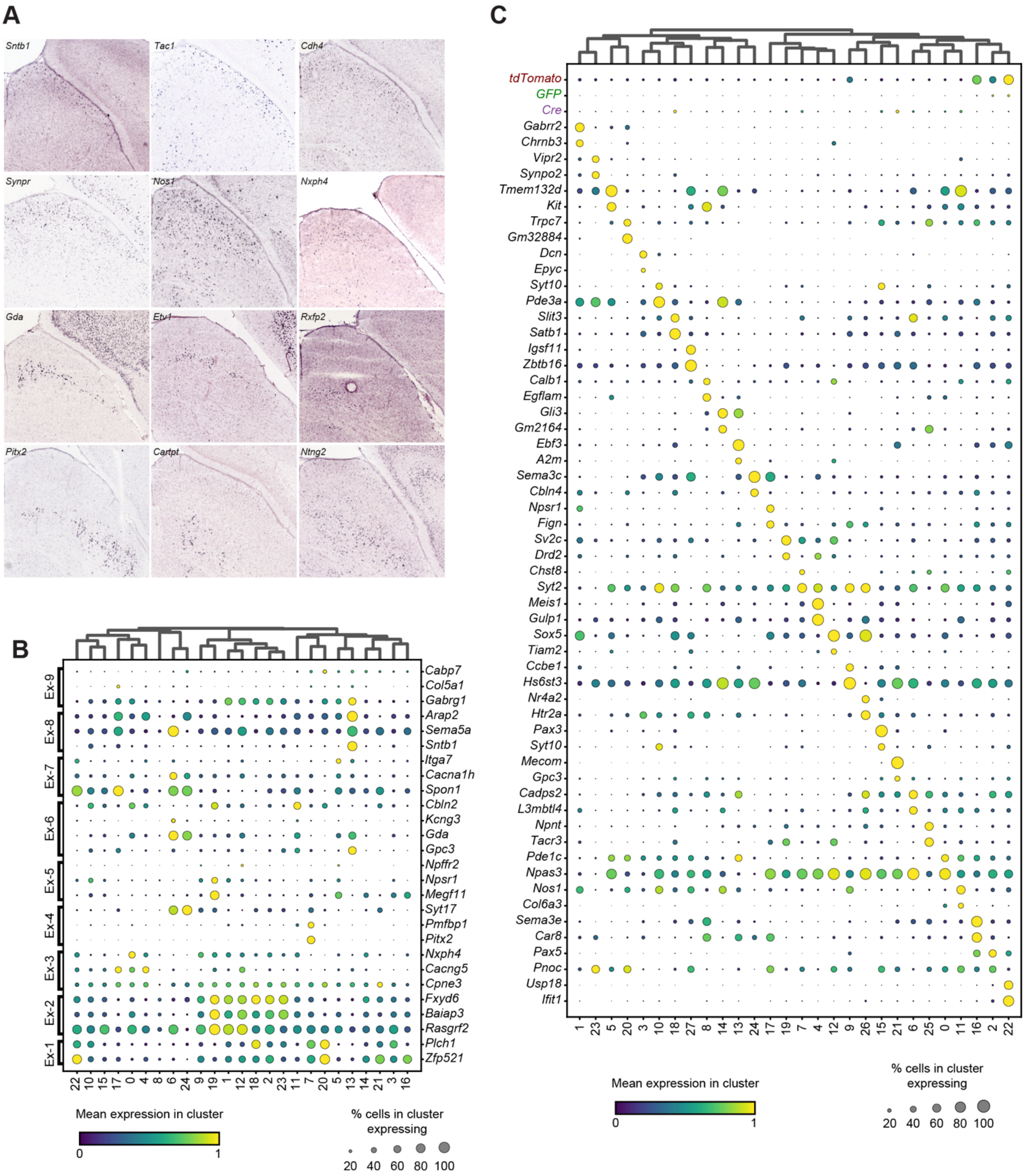

Figure S4. Further characterization of SC cell types and marker expression. Related to Figure 3. (A) Laminar distribution of markers for SC excitatory neuron subtypes. Images from Allen Mouse Brain Atlas *in situ* hybridization database (Lein et al., 2007). (B) Distribution of markers for SC excitatory subtypes described by Xie et al. (Xie et al., 2021) in SC excitatory clusters defined in this study. (C) Enriched and differentially expressed genes in SC inhibitory cell types and distribution of virally encoded transgenes.

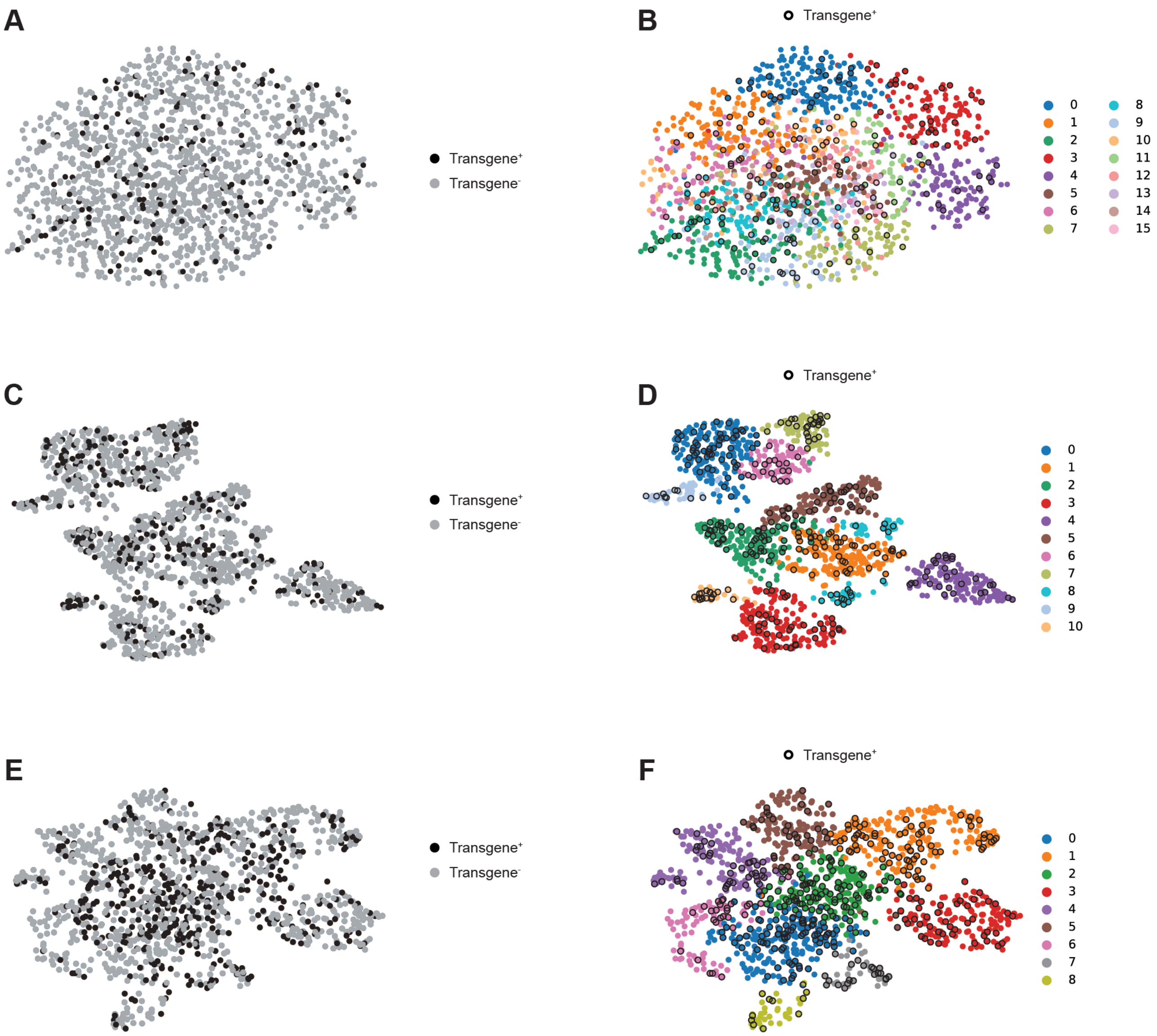

Figure S5. Analysis of the effects of viral transgene expression on endogenous gene expression within clusters in SC. Related to Figure 3. (A) Within cluster 7, transgene+ and transgene- cells are interspersed, suggesting that viral infection does not systematically perturb gene expression within these cells. (B) After finely subclustering cluster 7, transgene+ (black outlines) and transgene- cells are interspersed in each subcluster. (C)-(F) as in (A) and (B) for clusters 10 and 11.

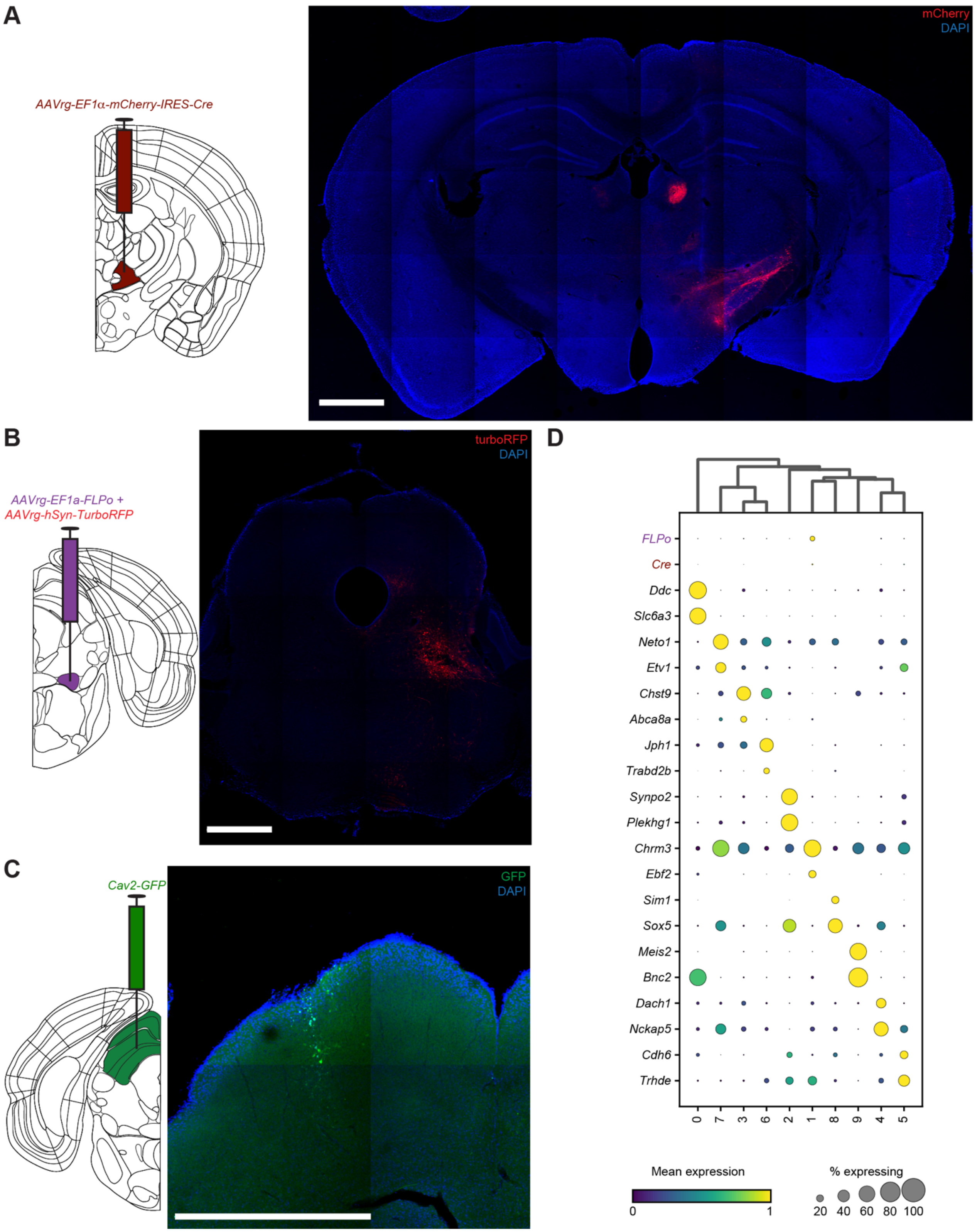

Figure S6. Histology of ventral midbrain injection sites and clustering of excitatory cell types. Related to Figure 5. (A) Injection site in LP. Injection track is visible. Labeling is also observed in ZI as well as lateral habenula, which projects to VM (Herkenham, 1979; Herkenham and Nauta, 1979). (B) Injection site in MLR. (C) Injection site in SC. Only a few SC cells expressed GFP and all were localized along the injection track. Expression was not observed in other structures projecting to SC, including cortex and contralateral SC. (D) Enriched and differentially

expressed genes in ventral midbrain excitatory cell types and distribution of virally encoded transgenes. Scale bars, 1 mm.

Transgenes	Number of expressing cells	Number of reads	Abundance in expressing cells
tdTomato	1,927	7,354	0.15 +/- 0.31
FLPo	575	1,276	0.10 +/- 0.12
mCherry-IRES-Cre	299	639	0.11 +/- 0.13
Endogenous Genes			
Snap25	8,053	19,143	0.08 +/- 0.05
Rbfox3	5,362	8,717	0.07 +/- 0.05
Slc17a6	1,648	2,249	0.05 +/- 0.04
Camk2a	3,137	4,521	0.06 +/- 0.05
Gad1	1,431	1,899	0.05 +/- 0.04
Gad2	2,581	4,288	0.07 +/- 0.05
Mog	236	325	0.13 +/- 0.08
Flt1	15	31	0.24 +/- 0.3

Table S3: Transgene detection in ventral midbrain sequencing dataset. Related to Figure 5. First column lists transgenes and endogenous genes analyzed. Second column indicates the number of cells in which each transgene or endogenous gene was detected. Third column indicates total number of reads corresponding to transgenes or endogenous genes in ventral midbrain sequencing dataset. Fourth column indicates relative expression of transgenes and common marker genes. To control for differences in abundance of different cell types, values denote the mean percentage of reads +/- standard deviation corresponding to a given marker or transgene in cells positive for that marker or transgene.

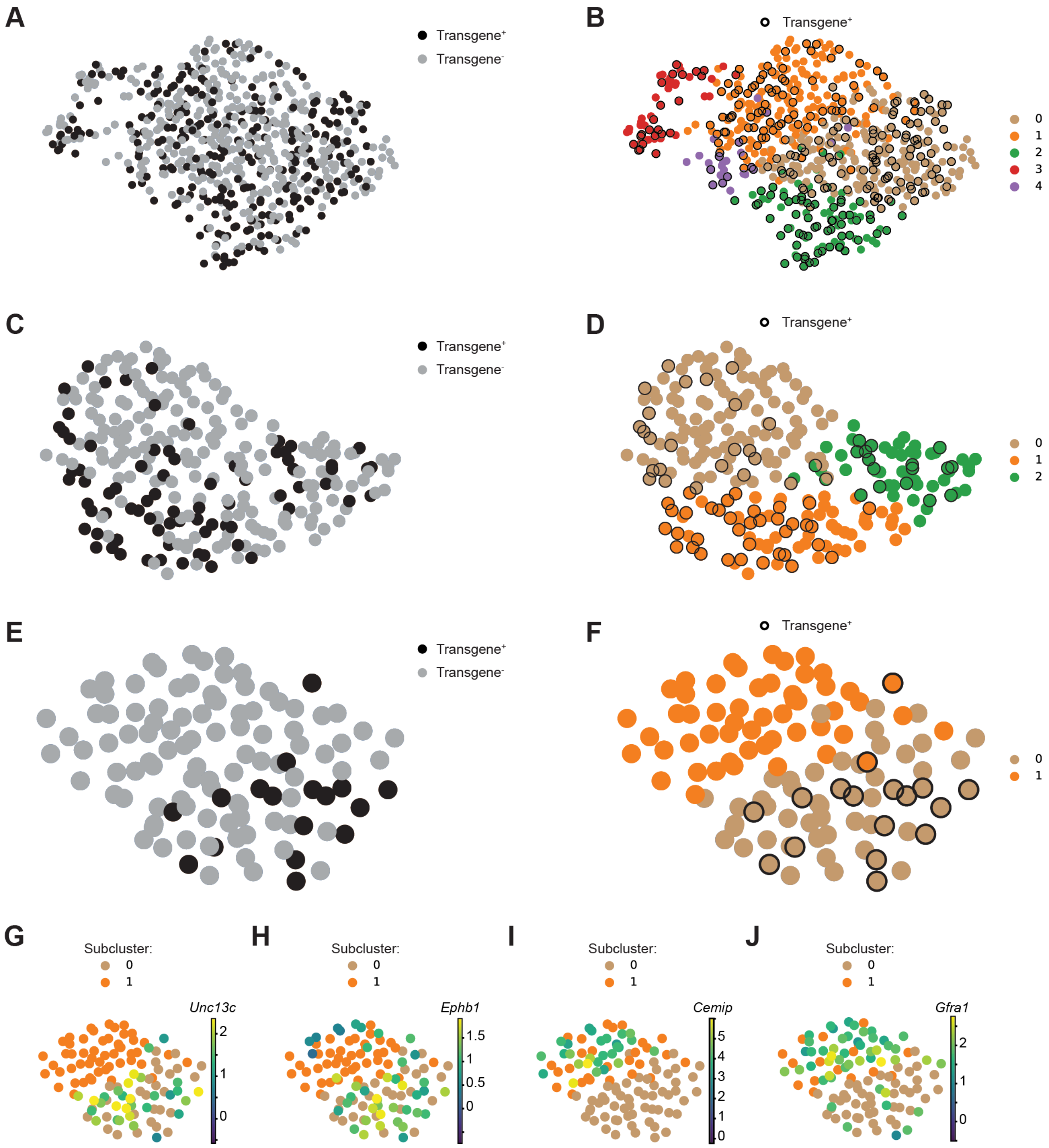

Figure S7. Analysis of the effects of viral transgene expression on endogenous gene expression within clusters in ventral midbrain. Related to Figure 5. Transgene$^+$ and transgene$^-$ cells are interspersed in clusters 2 (A), 5 (C), and 10 (E) from Figure 5, suggesting that the viral infection does not systematically perturb endogenous gene expression. This interspersion of transgene$^+$ and transgene$^-$ cells persists after finely subclustering clusters 2 and 5 (B, D), further indicating that variation in gene expression within these clusters are not linked to viral infection. Interestingly, although transgene$^+$ cells are interspersed with transgene$^-$ cells in cluster 10 (E), indicating that the transgenes did not disrupt viral transgene expression, transgene$^+$ cells are enriched in one half of cluster 10. Subclustering of cluster 10 (F) yields two populations, one of which contains nearly all the transgene$^+$ cells, suggesting that cluster 10 may comprise two closely related subtypes of which only one projects to VM. (G)-(J) Consistent with the prediction that cluster 10 comprises two closely related but distinct populations, a few endogenous genes are differentially expressed in these two subclusters. *Unc13c* and *Ephb1* are enriched in subcluster 0, whereas *Cemip* and *Gfra1* are enriched in subcluster 1. Scale bars indicate relative expression.
